# CWF19L2 is Essential for Male Fertility and Spermatogenesis by Regulating Alternative Splicing

**DOI:** 10.1002/advs.202403866

**Published:** 2024-06-18

**Authors:** Shiyu Wang, Yuling Cai, Tongtong Li, Yan Wang, Ziyou Bao, Renxue Wang, Junchao Qin, Ziqi Wang, Yining Liu, Zhaojian Liu, Wai‐Yee Chan, Xiangfeng Chen, Gang Lu, Zi‐Jiang Chen, Tao Huang, Hongbin Liu

**Affiliations:** ^1^ State Key Laboratory of Reproductive Medicine and Offspring Health Center for Reproductive Medicine Institute of Women Children and Reproductive Health Shandong University Jinan Shandong 250012 China; ^2^ National Research Center for Assisted Reproductive Technology and Reproductive Genetics Shandong University Jinan Shandong 250012 China; ^3^ Key Laboratory of Reproductive Endocrinology (Shandong University) Ministry of Education Jinan Shandong 250012 China; ^4^ Shandong Technology Innovation Center for Reproductive Health Jinan Shandong 250012 China; ^5^ Shandong Provincial Clinical Research Center for Reproductive Health Jinan Shandong 250012 China; ^6^ Shandong Key Laboratory of Reproductive Medicine Shandong Provincial Hospital Affiliated to Shandong First Medical University Jinan Shandong 250012 China; ^7^ Research Unit of Gametogenesis and Health of ART‐Offspring Chinese Academy of Medical Sciences Jinan Shandong 250012 China; ^8^ Key Laboratory of Experimental Teratology Ministry of Education Department of Cell Biology School of Basic Medical Sciences Cheeloo College of Medicine Shandong University Jinan Shandong 250012 China; ^9^ Advanced Medical Research Institute Shandong University Jinan Shandong 250012 China; ^10^ CUHK‐SDU Joint Laboratory on Reproductive Genetics School of Biomedical Sciences the Chinese University of Hong Kong Hong Kong 999077 China; ^11^ Shanghai Key Laboratory for Assisted Reproduction and Reproductive Genetics Shanghai 200000 China; ^12^ Department of Reproductive Medicine Ren Ji Hospital Shanghai Jiao Tong University School of Medicine Shanghai 200000 China

**Keywords:** CWF19L2, fertility, spermatogenesis, spermatogonial differentiation, splicing factor

## Abstract

The progression of spermatogenesis along specific developmental trajectories depends on the coordinated regulation of pre‐mRNA alternative splicing (AS) at the post‐transcriptional level. However, the fundamental mechanism of AS in spermatogenesis remains to be investigated. Here, it is demonstrated that CWF19L2 plays a pivotal role in spermatogenesis and male fertility. In germline conditional *Cwf19l2* knockout mice exhibiting male sterility, impaired spermatogenesis characterized by increased apoptosis and decreased differentiated spermatogonia and spermatocytes is observed. That CWF19L2 interacted with several spliceosome proteins to participate in the proper assembly and stability of the spliceosome is discovered. By integrating RNA‐seq and LACE‐seq data, it is further confirmed CWF19L2 directly bound and regulated the splicing of genes related to spermatogenesis (*Znhit1*, *Btrc*, and *Fbxw7*) and RNA splicing (*Rbfox1*, *Celf1*, and *Rbm10*). Additionally, CWF19L2 can indirectly amplify its effect on splicing regulation through modulating RBFOX1. Collectively, this research establishes that CWF19L2 orchestrates a splicing factor network to ensure accurate pre‐mRNA splicing during the early steps of spermatogenesis.

## Graphical abstract

Through germline conditional *Cwf19l2* mice exhibiting male sterility, CWF19L2 participate in the proper assembly and stability of the spliceosome, and CWF19L2 directly regulated the splicing of genes related to spermatogenesis and RNA splicing and indirectly amplify effect on splicing regulation through modulating RBFOX1 is demonstrated. Therefore, CWF19L2 functions in spermatogenesis and male fertility by orchestrating a splicing factor network to ensure accurate pre‐mRNA splicing.

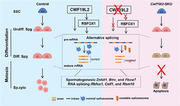

## 1 Introduction

The production of haploid spermatozoa is a highly intricate and meticulously orchestrated process involving diverse hormones, growth factors, and complex signal transduction pathways.^[^ ^1^ ^]^ Spermatogenesis is a crucial development pathway in adult animals, unfolding through three main processes: self‐renewal of spermatogonia stem cells (SSCs), differentiation of spermatogonia, and meiosis of spermatocytes. This intricate process begins with a diploid SSC, which divides into two spermatocytes and subsequently matures into four highly polarized spermatozoa.^[^ ^2^, ^3^, ^4^ ^]^ Given the consisting of diverse cell types and biological processes, spermatogenesis is an excellent model for studying gene regulation at the transcriptional and post‐transcriptional levels.^[^ ^5^ ^]^ Alternative splicing (AS) is an essential and universal mechanism to achieve post‐transcriptional regulation,^[^ ^6^ ^]^ which significantly enriches the transcriptomic and proteomic diversity within organisms, particularly the highly complex organs like the testis and brain.^[^ ^5^, ^6^, ^7^, ^8^ ^]^

Nascent precursor messenger RNA (pre‐mRNA) splicing consists of exon ligation and intron removal, and thus, these mRNAs have the potential to encode alternative protein isoforms.^[^ ^3^, ^6^, ^9^, ^10^ ^]^ This process is mediated by spliceosome proteins, including the large ribonucleoprotein (RNP) complex termed the spliceosome and specific splicing factors that facilitate the regulation of spliceosome and selection of splice site.^[^ ^9^, ^11^ ^]^ The intricate choreography of spliceosome assembly and splicing occurs within the specialized nuclear domains known as nuclear speckles.^[^ ^12^ ^]^ The entire pre‐mRNA splicing unfolds in four sequential stages of the spliceosome: assembly, activation, catalysis, and disassembly.^[^ ^12^, ^13^, ^14^ ^]^ These stages are further divided into ten central functional states regarding distinct spliceosome composition.^[^ ^12^, ^13^ ^]^ The exons are fully ligated in the previous eight steps, and ligated exons are released in the ninth step, thus forming the post‐catalytic (P) complex. In the tenth step, the intron lariat spliceosome (ILS) complex disassembles into the removed intron and the recycled spliceosome proteins.^[^ ^11^, ^12^, ^13^, ^14^ ^]^ With increasing interest in how both RNA splicing regulators and mRNA isoforms are modulated during spermatogenesis,^[^ ^5^, ^6^ ^]^ a growing number of novel splicing regulators involved in spermatogenesis continue to emerge through studies in mouse knockout models, such as BCAS2, BUD31, DDX5, hnRNPH1, RBM46, SAM68, SRSF1, SRSF2 and SRSF10,^[^ ^15^, ^16^, ^17^, ^18^, ^19^, ^20^, ^21^, ^22^, ^23^, ^24^ ^]^ suggesting that depletion of core splicing factors can alter the splicing of different subsets of genes and ultimately fertility. For example, RBM46 can bind to the mRNAs encoding multiple meiotic cohesion subunits and repress translation. Spermatocytes of *Rbm46* knockout mice exhibit disrupted synapses and meiotic arrest.^[^ ^23^ ^]^ BUD31, a component of the B^act^ complex, has an essential role in both SSCs pool maintenance and the initiation of spermatogenesis by mediating alternative splicing events (ASEs).^[^ ^24^ ^]^ Despite these insights, the underlying mechanisms of ASEs in spermatogenesis remain largely elusive.

CWF19‐like protein 2 (CWF19L2), evolutionarily conserved from *Saccharomyces cerevisiae* to humans, is a cell cycle control factor located in the nucleus and is speculated to be involved in mRNA splicing via the spliceosome.^[^ ^25^, ^26^, ^27^ ^]^ DRN1, the homolog of CWF19L2 in *S. cerevisiae*, has been confirmed to promote efficient intron turnover during splicing.^[^ ^27^ ^]^ Similarly, CWF19 in *S. pombe*, a homolog of CWF19L2, copurifies with the NineTeen Complex components of spliceosome.^[^ ^25^, ^28^ ^]^ In mammals, like yeast, CWF19L2 contains positively charged surface residues clamping over the lariat junction in the N‐terminus, and the CwfJ domain linked with HIT‐like domain by an extended bridging ɑ‐helix in the C‐terminus, which together binds the BPS/U2 duplex and the 3′‐tail of the intron.^[^ ^11^, ^29^ ^]^ Thus, CWF19L2 plays an essential role as a splicing factor in the last stage of spliceosome, when it is recruited into the ILS complex and forms extensive interactions with PRPF8 and the intron lariat.^[^ ^11^, ^14^ ^]^ In addition, CWF19L2 appeared in a screen for chromosomal deletion sites in breast carcinomas with other cell‐cycle regulation proteins.^[^ ^26^ ^]^ It is associated with Bardet‐Biedl syndrome IV, characterized by intellectual disabilities, obesity, and gonadal dysplasia. In addition, it may also interact with BRCA1 and BRCA2 and be a prospective target of ursolic acid in cancer therapy.^[^ ^30^, ^31^, ^32^ ^]^ Despite considerable insights into its structural and mechanistic aspects, its physiological function and potential mechanism in the reproductive system remain an intriguing area for future investigation.

In this study, we elucidate the critical role of CWF19L2 in spermatogenesis. Our findings demonstrate that deletion of CWF19L2 in male germ cells has negative effects on spliceosome stability and the AS of essential genes required for alternative splicing (*Rbfox1, Rbm10*, and *Celf1*) and spermatogenesis (*Znhit1*, *Btrc*, and *Fbxw7*), which are also involved in fertility and mutations of which ultimately lead to male sterility. In addition, CWF19L2 controls the AS of splicing factors like *Rbfox1* to achieve amplified splicing regulation. Our data position CWF19L2 as a fundamental component of the spliceosome and establish its paramount importance in regulating AS during spermatogenesis and the substantial impact on male fertility.

## 2 Results

### 2.1 CWF19L2 is Highly Expressed in Germ Cells During Spermatogenesis

**Figure 1:**
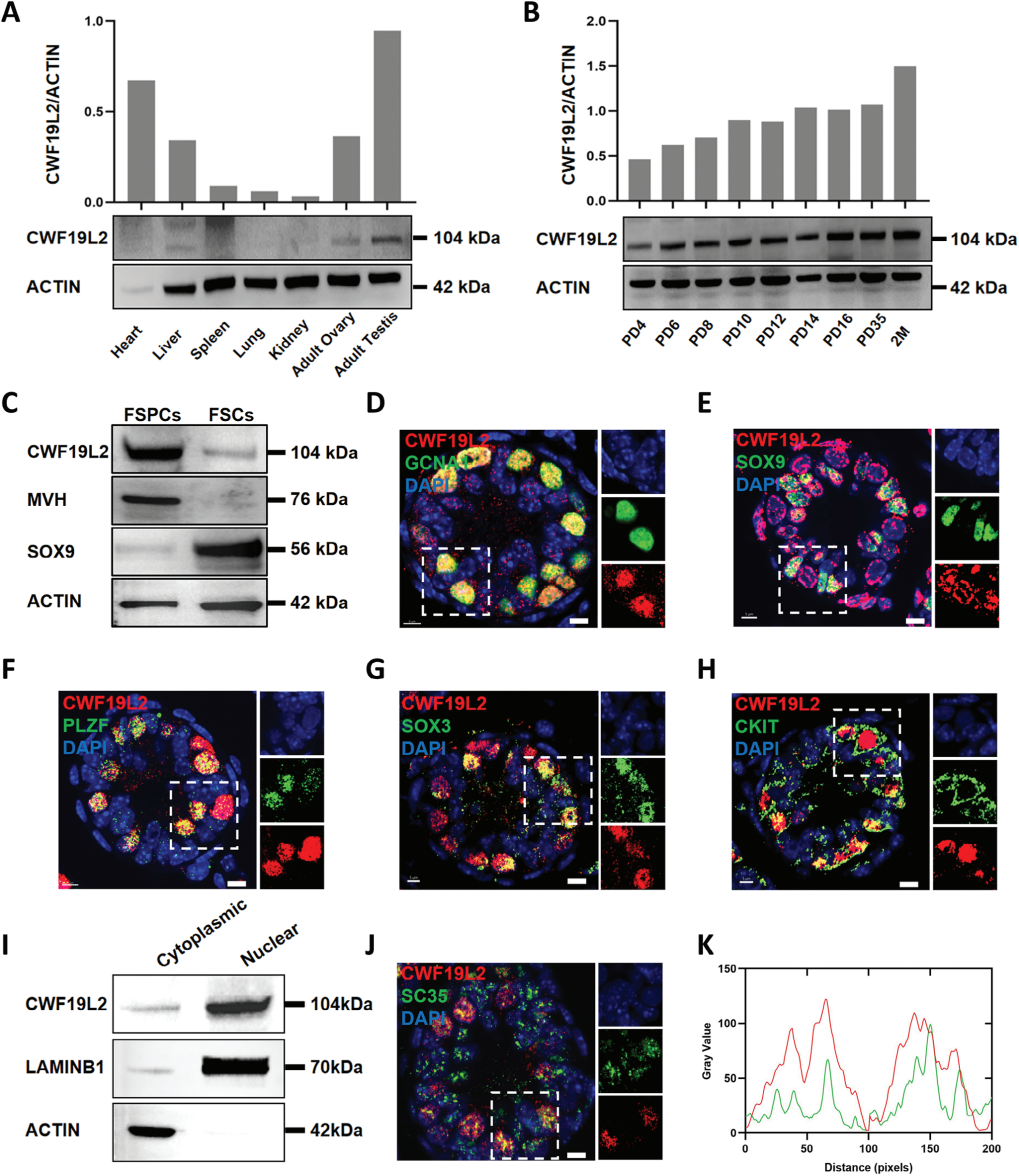
CWF19L2 is highly expressed in germ cells during spermatogenesis. A) Immunoblotting analysis of CWF19L2 in multiple adult wild‐type (WT) mice organs. ACTIN served as the loading control. Quantitative data are shown in the upper panel. B) Immunoblotting analysis of CWF19L2 at different developmental stages in mouse testes. Actin served as the loading control. Quantitative data are shown in the upper panel. C) Immunoblotting analysis of CWF19L2 in fractions of spermatogenic cells (FSPCs) and fractions of somatic cells (FSCs) in PD10 testes. The germ cell marker MVH and the Sertoli cell marker SOX9 were used as indicators of enrichment efficiency, and ACTIN served as the loading control. D) Co‐immunofluorescence staining of the germ cell marker GCNA1 (green) with CWF19L2(red) in testis sections of PD10 WT mice. DNA was stained with DAPI. Scale bars = 5 µm. E) Co‐immunofluorescence staining of the Sertoli cell marker SOX9 (green) with CWF19L2 (red) in testis sections of PD10 WT mice. DNA was stained with DAPI. Scale bars = 5 µm. F) Co‐immunofluorescence staining of the undifferentiated spermatogonia marker PLZF (green) with CWF19L2 (red) in testis sections of PD10 WT mice. DNA was stained with DAPI. Scale bars = 5 µm. G) Co‐immunofluorescence staining of the progenitor spermatogonia marker SOX3 (green) with CWF19L2(red) in testis sections of PD10 WT mice. DNA was stained with DAPI. Scale bars = 5 µm. H) Co‐immunofluorescence staining of the differentiated spermatogonia marker CKIT (green) with CWF19L2 (red) in testis sections of PD10 WT mice. DNA was stained with DAPI. Scale bars = 5 µm. I) Immunoblotting analysis of CWF19L2 in the nucleus and cytoplasm of spermatogenic cells from PD10 testes. ACTIN and LAMINB1 were used to indicate the separation efficiency of nuclear and cytoplasmic proteins, respectively. J) Co‐immunofluorescence staining of the nuclear speckles marker SC35 (green) with CWF19L2 (red) in testis sections of PD10 WT mice. DNA was stained with DAPI. Scale bars = 5 µm. K) mageJ was used to quantitatively analyze the intensity profiles within the white squares in J). The fluorescence intensity of SC35 was represented in green, and fluorescence intensity of CWF19L2 was represented in red.

CWF19L2 is evolutionarily conserved with identical C‐terminal CwfJ domain organization from yeast to humans (Figure S1A, Supporting Information), suggesting the common function across metazoans. To further investigate the role of *Cwf19l2* in mammals, we searched the expression data from the Expression Atlas,^[^ ^33^ ^]^ revealing approximate mRNA expression level in multiple organs (Figure S1B, Supporting Information). However, immunoblotting of various organs revealed a higher protein expression level in adult testis (**Figure** **1**A), which was potentially attributable to heightened AS frequencies in the testis. Immunoblotting of multiple developmental stages of testes showed that the CWF19L2 protein level increased gradually from postnatal day 4 (PD4) onwards and reached the first stable level at PD10 when the first wave of spermatogonia completed differentiation and entered meiosis (Figure 1B). This indicates a possible involvement of CWF19L2 in the early phases of spermatogenesis.

Using a differential adhesion method, we found by immunoblotting that CWF19L2 was expressed more in the fraction of spermatogenic cells (FSPCs) than in the fraction of somatic cells (FSCs) (Figure 1C). Co‐immunofluorescence staining of CWF19L2 with GCNA1 (a germ cell marker) and SOX9 (a Sertoli cell marker) confirmed that CWF19L2 was expressed primarily in germ cells of both PD10 and adult testes (Figure 1D and E; Figure S1D and E, Supporting Information). Next, we found that CWF19L2 colocalized with PLZF (an undifferentiated spermatogonia marker) (Figure 1F; Figure S1F, Supporting Information), SOX3‐positive (a progenitor spermatogonia marker) (Figure 1G; Figure S1G, Supporting Information), c‐KIT (a differentiated spermatogonia marker) (Figure 1H; Figure S1H, Supporting Information), and SYCP3 (a spermatocyte marker) (Figure S1I, Supporting Information) in testes of PD10 and adult mice. The colocalization of CWF19L2 with spermatogonia, spermatocytes, and Sertoli cells was consistent with published scRNA‐seq data of male germ cells for *Cwf19l2* expression (Figure S1C, Supporting Information).^[^ ^34^, ^35^ ^]^ In addition, co‐immunofluorescence of CWF19L2 with MVH (a germ cell marker) and PLZF in human testis sections exhibited a comparable expression pattern in germ cells to that of mice (Figure S1J and K, Supporting Information). Subcellular localization analysis based on immunoblotting of isolated nuclear and cytoplasmic proteins highlighted nuclear enrichment of CWF19L2 (Figure 1I). Co‐immunofluorescence staining of CWF19L2 and SC35 (a nuclear speckles marker) (Figure 1J and K; Figure S1L, Supporting Information) further demonstrated a high percentage of the SC35 signal overlapped with signal from CWF19L2, indicating CWF19L2 was probably localized in nuclear speckles, where the spliceosome assembles and functions. These results collectively suggest that CWF19L2 is widely expressed throughout male germ cell development and may have a conserved role in the nucleus.

### 2.2 CWF19L2 Physically Interacts with Multiple Spliceosome Components in the Testis

**Figure 2:**
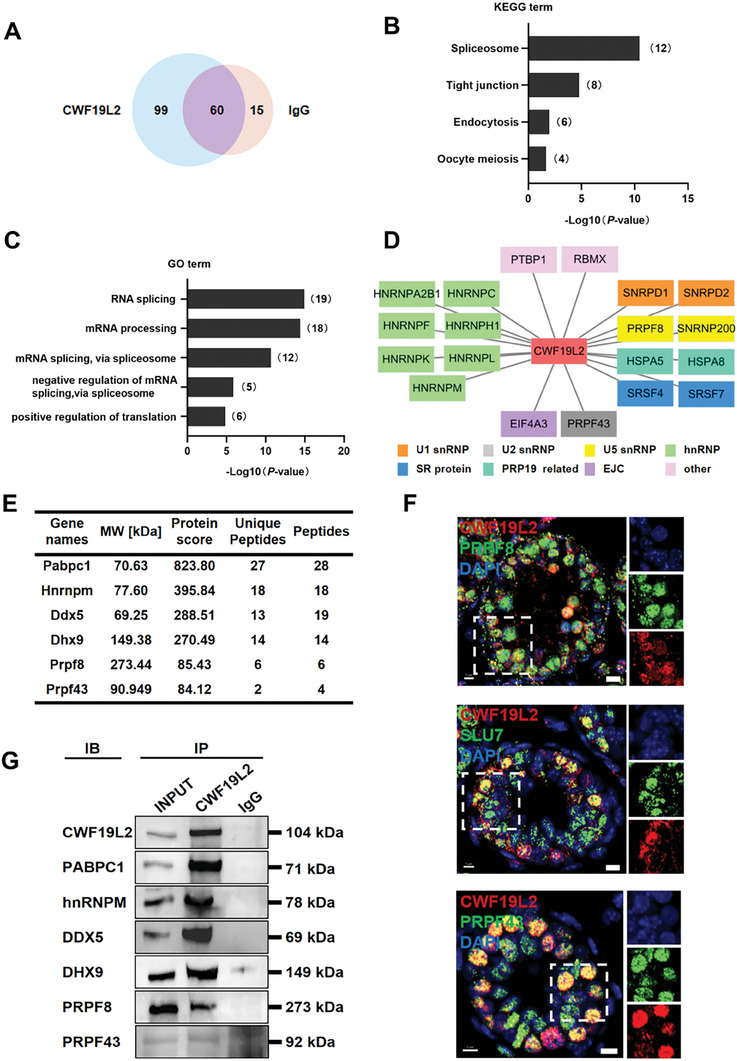
CWF19L2 physically interacts with multiple spliceosome components in testis. A) Venn diagram showing the 99 proteins interacting with CWF19L2 as detected by IP‐MS in PD10 WT testes. B) KEGG term enrichment analysis of the proteins that interact with CWF19L2 as identified in the IP‐MS data. C) GO term enrichment analysis of the interacting proteins of CWF19L2 identified from the IP‐MS data. D) The correlation network between CWF19L2 and splicing factors was constructed using Cytoscape. E) A list of proteins interacting with CWF19L2 in adult WT testes identified by IP‐MS is shown. F) Co‐immunofluorescence staining of CWF19L2 (red) with the spliceosome skeleton marker PRPF8, the P complex marker SLU7, the ILS complex marker PRPF43 (green) respectively in testis sections of PD10 *Cwf19l2*‐SKO and control mice. DNA was stained with DAPI. Scale bars = 5 µm. G) Validation of the interactions between CWF19L2 and putative CWF19L2‐interacting proteins (PABPC1, hnRNPM, DDX5, DHX9, PRPF8, and PRPF43) in PD10 testes by in vivo co‐immunoprecipitation (Co‐IP) assays. IgG was used as the negative control.

To elucidate the physiological functions of CWF19L2 in germ cell development, we used immunoprecipitation‐mass spectrometry (IP‐MS) to identify candidate CWF19L2‐interacting proteins in PD10 wild‐type (WT) testes. This analysis identified a total of 99 candidate proteins that interacted with CWF19L2 (**Figure** **2**A; Data 2, Supporting Information).

Kyoto Encyclopedia of Genes and Genomes (KEGG) analysis unveiled that CWF19L2 was predominantly associated with many spliceosome proteins (Figure 2B). Furthermore, Gene Ontology (GO) analysis highlighted that 19 of 99 candidates had RNA splicing‐related functions, and majority of them were spliceosome proteins such as hnRNP M, PRPF8, and PRPF43 (Figure 2C–E). Prior studies of pre‐mRNA splicing have indicated that SLU7 and the ligated exon dissociate from the P complex, and CWF19L2 and PRP43 are sequentially recruited into the ILS complex. PRPF8 functions as a structural protein of the spliceosome during the P‐to‐ILS transition to enable the attachment of the above proteins.^[^ ^11^, ^13^, ^14^ ^]^ We performed co‐immunofluorescence to verify the colocalization of CWF19L2 with PRPF8, SLU7, and PRPF43 in the nuclear speckles of spermatogonia (Figure 2F), suggesting the possibility of functional interplay between these proteins. Moreover, our findings indicated that CWF19L2 also interacted with some RNA processing proteins, like PABPC1, DDX5, and DHX9 (Figure 2C,E), which were previously demonstrated to be essential for spermatogenesis,^[^ ^17^, ^36^, ^37^ ^]^ further underscoring the likely essential function of CWF19L2 in these intricate processes.

To confirm these interactions, we subsequently performed CWF19L2 co‐immunoprecipitation (Co‐IP) with extracts from PD10 WT testes, followed by immunoblotting, which verified the interaction of CWF19L2 with PABPC1, hnRNP M, DDX5, DHX9, PRPF8, PRPF43 (Figure 2G). These findings indicate that CWF19L2 cooperated with several spliceosome proteins and some spermatogenesis‐related RNA processing factors in the testis, implying a critical role for CWF19L2 in these fundamental biological processes.

### 2.3 CWF19L2 is Essential for Spermatogenesis and Male Fertility

**Figure 3:**
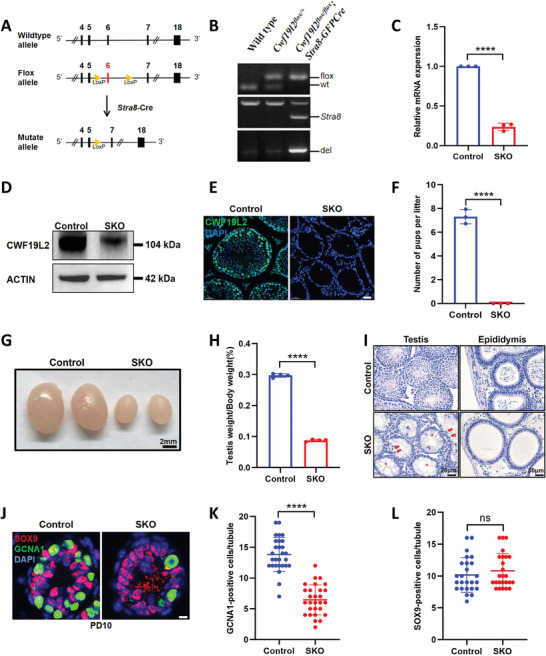
CWF19L2 is essential for spermatogenesis and male fertility. A) The schematic showing the insertion of loxp sites to flank exon 6 of the mouse *Cwf19l2* gene and deletion of *Cwf19l2* in germ cells using *Stra8*‐Cre. B) PCR‐based genotype of the PD5 mouse tails is shown. C) QPCR analysis of knockout efficiency of *Cwf19l2* in testes of adult *Cwf19l2*‐SKO and control mice. Data are presented as mean ± SD, n = 3, ****P < 0.0001 by two‐tailed Student’ s *t*‐test. D) Immunoblotting analysis of the knockout efficiency of CWF19L2 in the testes of adult *Cwf19l2*‐SKO and control mice. ACTIN served as the loading control. E) Immunofluorescence staining analysis of the knockout efficiency of CWF19L2 (green) in testis sections of adult *Cwf19l2*‐SKO and control mice. DNA was stained with DAPI. Scale bars = 30 µm. F) Fertility tests of adult *Cwf19l2*‐SKO and control mice showed the cumulative number of pups per mouse. Data are presented as mean ± SD, n = 3, ****P < 0.0001 by two‐tailed Student’ s *t*‐test. G) Gross morphology of the testes of adult *Cwf19l2*‐SKO and control mice. Scale bars = 2 mm. H) The ratio of testis weight to body weight in adult *Cwf19l2*‐SKO and control mice. Data are presented as the mean ± SD, n = 4, ****P < 0.0001 by two‐tailed Student’ s *t*‐test. I) Hematoxylin staining of testes (top panel, Scale bars = 20 µm) and epididymides (ep) (bottom panel, Scale bars = 50 µm) of adult *Cwf19l2*‐SKO and control mice. Arrows, apoptotic spermatocytes; asterisks, empty seminiferous tubules. J) Co‐immunofluorescence staining of GCNA1 (green) with SOX9 (red) in testis sections of PD10 *Cwf19l2*‐SKO and control mice. DNA was stained with DAPI. Scale bars = 5 µm. K) The quantification of GCNA1‐positive cells per tubule in J). Data are presented as the mean ± SD, ****P < 0.0001 by two‐tailed Student’ s *t*‐test. L) The quantification of SOX9‐positive cells per tubule in (). Data are presented as the mean ± SD, ns: not significant by two‐tailed Student’ s *t*‐test.

To investigate the functional role of CWF19L2 in spermatogenesis, we generated mice harboring a floxed *Cwf19l2* allele, where exon 6 was flanked by loxP sites. By crossing *Cwf19l2*‐floxed mice with *Stra8‐GFPCre* transgenic mice (**Figure** **3**A), we created germline conditional *Cwf19l2* knockout mice (*Cwf19l2^flox/flox^; Stra8‐GFPCre*, “*Cwf19l2‐*SKO” in the text or “SKO” in the figures). *Stra8‐GFPCre* begins to function as early as PD3 in type A1 spermatogonia through preleptotene stage spermatocytes in males.^[^ ^38^ ^]^ The genotype of the conditional knockout mice was confirmed by PCR of tail biopsy DNA at PD5 (Figure 3B). Additionally, qPCR, immunoblotting, and immunofluorescence of testes all supported the expected reductions in mRNA and protein levels in *Cwf19l2‐*SKO compared to controls (*Cwf19l2^flox/flox^* or *Cwf19l2^flox/+^; Stra8‐GFPCre*, “control” hereafter) (Figure 3C–E). Germ cells from *Cwf19l2*‐SKO and control mice revealed significant knockout of CWF19L2 (Figure S2A, Supporting Information), but somatic cells showed unchanged expression level (Figure S2B, Supporting Information), suggesting the residual CWF19L2 protein in testes may mainly originate from supporting cells.

Although *Cwf19l2‐*SKO males appeared normal and viable, six‐month‐long fertility tests showed that they were sterile (Figure 3F). The testis size of *Cwf19l2‐*SKO mice was considerably smaller than their littermate controls (Figure 3G), and the testis/body weight ratio of *Cwf19l2‐*SKO mice was significantly reduced compared to controls (Figure 3H). Histological examinations with hematoxylin staining showed a significant decrease in the number of germ cells, especially spermatocytes entering meiosis, and a pronounced increase in the number of apoptotic cells (the nucleus was deeply stained, the cytoplasm was condensed, and the chromatin was clumped) in the vacuolated seminiferous tubules of adult *Cwf19l2‐*SKO mice (Figure 3I). No mature sperm were observed in the epididymis of *Cwf19l2‐*SKO mice (Figure 3I).

Meanwhile, through similar breeding strategy, *Cwf19l2* ^flox/flox^ mice also crossed with *Amh‐Cre* mice, specifically expressing in the Sertoli cells,^[^ ^39^ ^]^ to generate the *Cwf19l2^flox/flox^*; *Amh‐Cre* mice (“*Cwf19l2*‐AKO” in the text or “AKO” in the figures) (Figure S2C, Supporting Information). Phenotypically, *Cwf19l2*‐AKO male mice exhibited normal viability and fertility despite smaller testes (Figure S2D, Supporting Information) and a lower testis/body weight ratio (Figure S2E, Supporting Information) compared to littermate controls. Histological analysis showed normal spermatogenesis including all stages of spermatogonia, spermatocytes, and spermatozoa in testes and morphologically normal sperm in the epididymis (Figure S2F, Supporting Information). A few apoptotic cells and 6%–8% atrophied seminal tubules were observed in the testes of PD20 *Cwf19l2*‐AKO mice (Figure S2G and H, Supporting Information), which could at least partially account for the smaller testes of adult mice, but no effect on fertility. Therefore, the above results suggest that CWF19L2 plays a more essential role in germ cells than in Sertoli cells.

The time point of apoptosis was determined by the TUNEL assay, and a large number of TUNEL‐positive cells were found surrounding the time of meiosis initiation at PD8 and PD10 in *Cwf19l2‐*SKO mice (Figure S3A and B, Supporting Information). The persistence of this massive level of apoptosis indicates that each round of spermatogenesis was negatively impacted by CWF19L2 deficiency. We further explored the cell types undergoing apoptosis by co‐immunofluorescence staining of GCNA1 and SOX9 at PD6, PD8, and PD10 (Figure 3J; Figure S3E,H, Supporting Information). We found a gradual reduction in germ cells in *Cwf19l2‐*SKO testes from PD6 onwards (Figure 3K; Figure S3F,I, Supporting Information) while Sertoli cells were not significantly altered at these three time points (Figure 3L; Figure S3G,J, Supporting Information). The reduced expression of MVH (Figure S3C, Supporting Information) and the stable level of SOX9 (Figure S3D, Supporting Information) were further confirmed at the mRNA level between *Cwf19l2‐*SKO and control testes. These comprehensive findings indicate that CWF19L2 has an indispensable role in germ cell development and that deletion of CWF19L2 causes male sterility.

### 2.4 Ablation of CWF19L2 Causes Aberrant mRNA Splicing in Male Germ Cells

**Figure 4:**
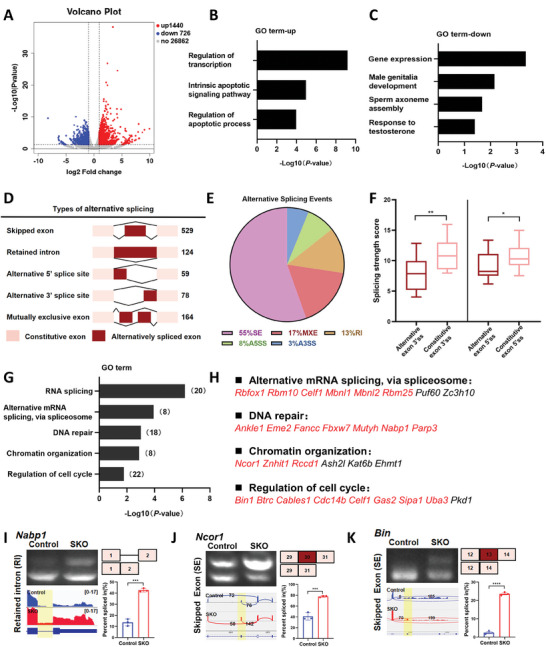
Ablation of CWF19L2 causes aberrant mRNA splicing in male germ cells. A) Volcano plot of DEGs determined by RNA‐seq analysis of PD6 *Cwf19l2*‐SKO mice compared to control mice. Blue dots represent significantly downregulated genes, red dots represent significantly upregulated genes, and gray dots represent unchanged genes. B) GO term enrichment analysis of the significantly upregulated genes in the RNA‐seq data. C) GO term enrichment analysis of the significantly downregulated genes in the RNA‐seq data. D) Schematic diagram of five AS types significantly affected by depletion of CWF19L2 in the RNA‐seq data. The numbers of predicted ASEs in each category are indicated. E) Pie chart depicting the proportions of different types of ASEs in the RNA‐seq data. F) Box plots of splice site scores calculated for CWF19L2‐regulated skipped exons. Horizontal line indicates the median and 25% to 75% bounds of the box. *P < 0.05, **P < 0.01 by two‐tailed Student’ s *t*‐test. G) GO term enrichment analysis of genes with abnormal ASEs in the RNA‐seq. The numbers of representative genes are indicated. H) GO term enrichment analysis of genes with abnormal ASEs in the RNA‐seq. Genes marked in red represent successful verification by RT‐PCR(I‐K) Visualization and validation of abnormal ASEs identified by RNA‐seq in sorted germ cells of *Cwf19l2*‐SKO and control mice. Gel images showing the RT‐PCR analysis of ASEs of the changed splicing genes in top‐left panel. Tracks from Integrative Genomics Viewer (IGV) for selected candidate genes are shown in the bottom‐left panel, and differentially spliced parts are shaded. Schematics of ASEs are shown in the top‐right panel. Changes in “percent spliced in (△PSI)” are shown in the bottom‐right panel. Data are presented as the mean ± SD, n = 3, ns: not significant, *P < 0.05, **P < 0.01, ***P < 0.001, ****P < 0.0001 by two‐tailed Student’ s *t*‐test.

TUNEL assay revealed no significant differences in apoptosis levels at PD4; however, by PD6, marked variations were observed (Figure S3A and B, Supporting Information). With consideration that CWF19L2 predominantly localized within the nucleus of germ cells (Figure 1J; Figure S1L, Supporting Information) and interacted with several vital spliceosome proteins (Figure 2G), we conducted RNA‐seq analysis of PD4 and PD6 testes from *Cwf19l2*‐SKO and control mice to better understand the effects of CWF19L2 on alternative splicing regulation in male germ cells. The findings confirmed the relatively minor discrepancies at PD4 (Figure S4A, Supporting Information), and further supported the more pronounced differences we observed at PD6 (**Figure** **4**A).

Through analyzing differentially expressed genes (DEGs) identified by RNA‐seq at PD6, we observed that deletion of CWF19L2 resulted in the upregulation of 1440 genes (66.4%) and downregulation of 728 genes (33.6%) (*P* < 0.05, |log2FoldChange| > 1.0) (Figure 4A and Data 3, Supporting Information). GO analysis showed that the upregulated genes were related to the regulation of transcription like *Tnf*, and *Pou2f2* and regulation of apoptosis like *Bax* (Figure 4B; Figure S4B and C, Supporting Information) and that the downregulated genes were involved in male genitalia development like *Shh*, and sperm axoneme assembly like *Drc7* (Figure 4C, Figure S4D and E, Supporting Information). These results suggest that CWF19L2 mainly regulates vital cellular processes like transcription and apoptosis to support spermatogenesis.

The analysis of mRNA splicing changes in *Cwf19l2*‐SKO and control testes unveiled nearly 950 abnormal ASEs, including 529 skipped exons (SE), 124 retained introns (RI), 59 alternative 5′ splice sites (A5SS), 78 alternative 3′ splice sites (A3SS), and 164 mutually exclusive exons (MXE) (Figure 4D and Data 4, Supporting Information) (*P* < 0.05, |△PSI| > 10%), affecting ≈850 genes. Skipped exons were the predominant splicing type (55%) among the ASEs in *Cwf19l2*‐SKO mice (Figure 4E). We further analyzed splicing site strength of skipped exons and found that both alternative exon 3′ splice sites and alternative 5′ splice sites had lower splicing strength scores than those in constitutive exon 3′ splice sites and 5′ splice sites in *Cwf19l2*‐SKO mice (Figure 4F), suggesting that CWF19L2 may participate in splice site recognition to regulate pre‐mRNA splicing in testes. Utilizing GO analysis to investigate the ASEs further, we discovered that ASEs were involved in alternative mRNA splicing via spliceosome (*Rbfox1, Rbm10*, and *Celf1*) (Figure 4G). In addition, by comparing ASEs with list of spermatogenesis‐related genes, we screened out a series of genes including *Znhit1, Btrc*, *Rbm25* and *Fbxw7*.^[^ ^40^, ^41^, ^42^, ^43^ ^]^ We selected several ASEs, such as *Nabp1*, and ≈80% (24/30) could be validated by RT‐PCR (Figure 4H–K; Figure S4G, Supporting Information), thus confirming the reliability of the RNA‐seq data. Interestingly, we investigated the relationship between AS and gene expression and found a limited overlap of DEGs with ASEs, 51/2166 (2.4%), suggesting CWF19L2 may regulate splicing and transcription through potential distinct mechanisms (Figure S4F, Supporting Information).

Bulk RNA‐seq offered a broader overview, revealing not only the impact of CWF19L2 knockout in germ cells but also its subsequent effects on the testicular homeostasis. To gain a more precise understanding of CWF19L2's involvement in spermatogonia, we conducted SMART‐seq analysis on c‐KIT positive spermatogonia isolated from *Cwf19l2*‐SKO and control mice (Figure S5A–C, and Data 3, Supporting Information). Remarkably, our findings demonstrated significant congruence between SMART‐seq and RNA‐seq results. Specifically, 74% of DEGs and 66% of ASEs identified by SMART‐seq were also detected in the PD6 RNA‐seq (Figure S5D and E, Supporting Information). Additionally, Gene Ontology (GO) analysis of DEGs and ASEs in SMART‐seq yielded nearly identical results to those obtained from RNA‐seq (Figure 4B,C,G; Figure S5F–H, Supporting Information). Taken together, these analyses suggest that CWF19L2 plays a regulatory role in the splicing of critical genes associated with spermatogenesis and splicing mechanisms.

### 2.5 Absence of CWF19L2 Results in Impaired Spermatogonial Differentiation

**Figure 5:**
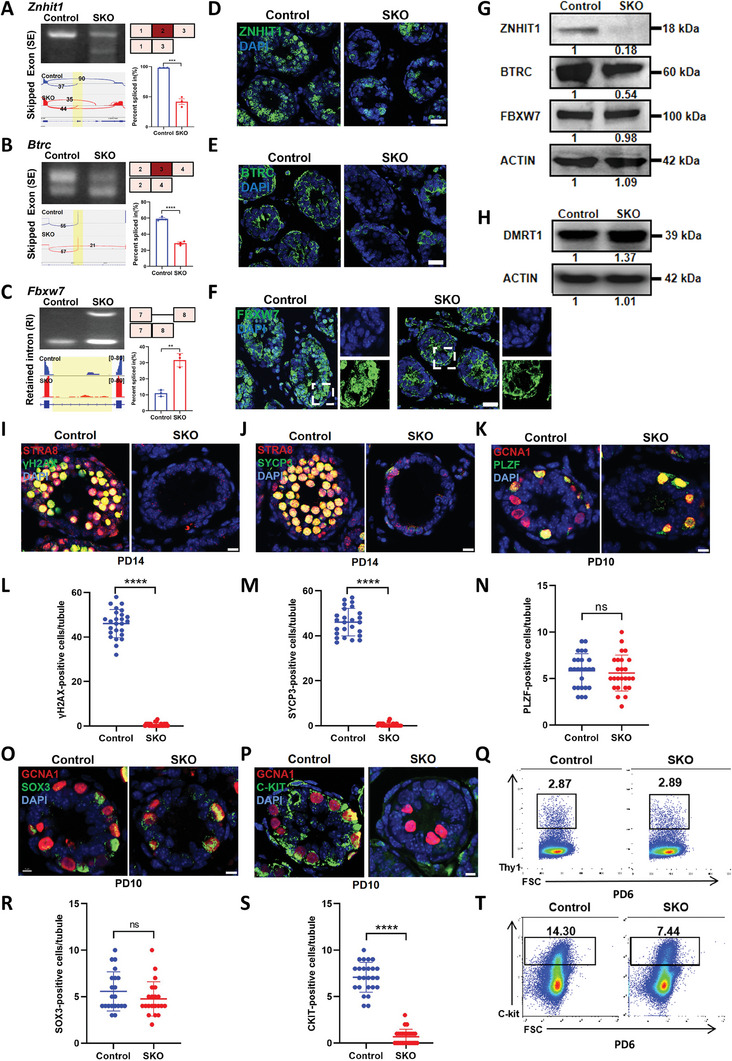
Absence of CWF1L2 results in impaired spermatogonial differentiation. A) Visualization and validation of *Znhit1* abnormal ASEs in sorted germ cells from *Cwf19l2*‐SKO and control mice. B) Visualization and validation of *Btrc* abnormal ASEs in sorted germ cells from *Cwf19l2*‐SKO and control mice. C) Visualization and validation of *Fbxw7* abnormal ASEs in sorted germ cells from *Cwf19l2*‐SKO and control mice. D) Immunofluorescence staining of ZNHIT1 (green) in testis sections of PD10 *Cwf19l2*‐SKO and control mice. DNA was stained with DAPI. Scale bars = 15 µm. E) Immunofluorescence staining of BTRC (green) in testis sections of PD10 *Cwf19l2*‐SKO and control mice. DNA was stained with DAPI. Scale bars = 15 µm. F) Immunofluorescence staining of FBXW7 (green) in testis sections of PD10 *Cwf19l2*‐SKO and control mice. DNA was stained with DAPI. Scale bars = 15 µm. G) Immunoblotting analysis of ZNHIT1, BTRC and FBXW7 protein in testes of PD10 *Cwf19l2*‐SKO and control mice. ACTIN served as the loading control. (H) Immunoblotting analysis of DMRT1 protein in testes of PD10 *Cwf19l2*‐SKO and control mice. ACTIN served as the loading control. I) Co‐immunofluorescence staining of STRA8 (red) with the meiotic marker γH2AX (green) in testis sections of PD14 *Cwf19l2*‐SKO and control mice. DNA was stained with DAPI. Scale bars = 5 µm. J) Co‐immunofluorescence staining of STRA8 (red) with SYCP3 (green) in testis sections of PD14 *Cwf19l2*‐SKO and control mice. DNA was stained with DAPI. Scale bars = 5 µm. K) Co‐immunofluorescence staining of GCNA1 (red) with PLZF (green) in testis sections of PD10 *Cwf19l2*‐SKO and control mice. DNA was stained with DAPI. Scale bars = 5 µm. L) The quantification of γH2AX‐positive cells per tubule in I). Data are presented as the mean ± SD, ****P < 0.0001 by two‐tailed Student’ s *t*‐test. M) The quantification of SYCP3‐positive cells per tubule in J). Data are presented as the mean ± SD, ****P < 0.0001 by two‐tailed Student’ s *t*‐test. N) The quantification of PLZF‐positive cells per tubule in K). Data are presented as the mean ± SD, ns: not significant by two‐tailed Student’ s *t*‐test. O) Co‐immunofluorescence staining of GCNA1 (red) with SOX3 (green) in testis sections of PD10 *Cwf19l2*‐SKO and control mice. DNA was stained with DAPI. Scale bars = 10 µm. P) Co‐immunofluorescence staining of GCNA1 (red) with c‐KIT (green) in testis sections of PD10 *Cwf19l2*‐SKO and control mice. DNA was stained with DAPI. Scale bars = 5 µm. Q) Flow cytometry analysis of the proportion of the spermatogonia stem cell marker THY1‐positive cells in the testes of PD6 *Cwf19l2*‐SKO and control mice. R) The quantification of SOX3‐positive cells per tubule in O). Data are presented as the mean ± SD, ns: not significant by two‐tailed Student’ s *t*‐test. S) The quantification of c‐KIT‐positive cells per tubule in P). Data are presented as the mean ± SD, ****P < 0.0001 by two‐tailed Student’ s *t*‐test. (T) Flow cytometry analysis of the proportion of c‐KIT‐positive cells in the testes of PD6 *Cwf19l2*‐SKO and control mice.

Given that the splicing of genes related to spermatogenesis was affected in the *Cwf19l2*‐SKO mice, we investigated the abnormal splicing of corresponding exons in isolated spermatogonia by RT‐PCR. For example, *Znhit1*, which regulates meiotic initiation,^[^ ^44^ ^]^ produced transcripts that skipped exon 2 (**Figure** **5**A). *Btrc*, involved in the mitosis‐meiosis transition via DMRT1 degradation,^[^ ^45^, ^46^ ^]^ tended to display shortened transcripts skipping exon 3 (Figure 5B). We detected the comparable mRNA expression of *Znhit1* and *Btrc* by qPCR (Figure S6A and B, Supporting Information), indicating that the genes are not likely affected at the transcriptional level, and the decreased protein levels of ZNHIT1 and BTRC by immunofluorescence and immunoblotting in *Cwf19l2*‐SKO testes compared to control (Figure 5D,E,G). Additionally, we detected an increase in DMRT1 protein in *Cwf19l2*‐SKO testes (Figure 5H; Figure S6G, Supporting Information). Differences in mRNA and protein expression may be due to nonsense‐mediated decay (NMD), which is typically triggered by the presence of premature termination codons (PTCs), including UAA, UAG, and UGA, and result in mRNA degradation.^[^ ^47^ ^]^ Alternative splicing is a known mechanism for introducing PTCs.^[^ ^48^ ^]^ Utilizing IGV, we identified several aberrant PTCs in the skipped exon 2 of *Znhit1* and exon 3 of *Btrc* in the RNA‐seq data (Figure S6C, Supporting Information). In addition, we designed primers targeting sequence upstream of the skipped exon to quantify transcripts lacking the exon (defined as “short”), as well as transcripts containing the skipped exon (defined as “long”; Figure S6D, Supporting Information) for qPCR analysis of their expression levels in c‐KIT positive spermatogonia sorted from *Cwf19l2*‐SKO and control mice. The short/long ratios of *Znhit1* and *Btrc* were both lower in *Cwf19l2*‐SKO mice compared to controls (Figure S6E and F, Supporting Information). These results suggested the presence of PTCs in these transcripts and possible activation of NMD. However, these ratios only decreased by ≈25% and 15% for *Znhit1* and *Btrc*, respectively, whereas protein expression decreased by 80% and 50%, suggesting there were still a possible post‐transcriptional regulatory mechanism.

In addition, we observed *Fbxw7* was retained in intron 7 (Figure 5C), whose deletion causes spermatogonia to fail to enter differentiation.^[^ ^40^, ^42^, ^43^ ^]^ However, abnormal transcripts did not always result in abnormal protein expression. Immunoblotting and immunofluorescence exhibited similar protein levels, but we found a distinct difference in the position of FBXW7 in *Cwf19l2*‐SKO testes (Figure 5F,G). Besides, the abnormal splicing of other spermatogenesis‐related genes, like *Rbm25*, *Tcf7*, *Tcf7l1*, *Eif4g1*, *Auts2*, *Pbrm1*, *Tmem131l*, *Cdip1* and *Ncapg* were also successfully confirmed (Figure S6H, Supporting Information), and we obtained decreased protein levels of RBM25, TCF7, and TCF7L1 (Figure S6I, Supporting Information). This suggested that CWF19L2 ablation may affect the spermatogenesis by regulating some spermatogenesis‐related gene splicing.

To assess the abnormality of germ cells in *Cwf19l2*‐SKO testis during spermatogenesis, we performed co‐immunofluorescence staining of STRA8 (a meiotic initiation marker) and pH3 (a mitotic and meiotic metaphase cell marker). Meiotic cells labeled by STRA8 were scarcely observed in PD10 and PD14 (already in meiosis) *Cwf19l2‐*SKO testes, while mitotic cells (pH3‐positive and STRA8‐negative) were comparable between *Cwf19l2*‐SKO and control (Figure S7A,B,D,E, Supporting Information). Subsequently, co‐immunofluorescence staining of STRA8 and γH2AX (a double‐strand break repair marker) was performed to further explore whether meiosis occurred normally in *Cwf19l2‐*SKO testes. In contrast to control, both STRA8‐ and H2AX‐positive cells were absent in *Cwf19l2*‐SKO testes (Figure 5I,L). Similar results were observed in co‐immunofluorescence staining of STRA8 and SYCP3 (a meiosis marker) (Figure 5J,M). Reduced mRNA expression of *Stra8*, *γH2ax*, and *Sycp3* was also evident by qPCR in sorted germ cells (Figure S7G, Supporting Information). Therefore, these results suggest that the absence of CWF19L2 could result in the failure of meiosis but did not affect early mitosis.

During the mitosis, germ cells decide whether or not to differentiate. To shed light on the role of CWF19L2 in spermatogonial differentiation, we employed the co‐immunofluorescence to analyze the germ cell fates in *Cwf19l2*‐SKO and control testes. PLZF‐positive spermatogonia were unaffected in PD10 and adult *Cwf19l2‐*SKO testes (Figure 5K,N; Figure S7C,F, Supporting Information), indicating that *Cwf19l2* deletion did not affect SSC self‐renewal. SOX3‐positive spermatogonia were also unaffected (Figure 5O,R), suggesting the normal fate of spermatogonia in *Cwf19l2‐*SKO mice to prepare for differentiation. However, c‐KIT‐positive spermatogonia were greatly reduced in *Cwf19l2*‐SKO testes (Figure 5P,S), indicating that spermatogonial differentiation was impaired. Consistent with this, we detected normal mRNA levels of SSC self‐renewal and maintenance genes (*Gfra1*, *Plzf*, *Lin28a*, and *Sall4*) (Figure S7H, Supporting Information), slightly reduced progenitor spermatogonia genes (*Sox3* and *Ngn3*) (Figure S7I, Supporting Information), and significantly decreased genes involved in spermatogonial differentiation (*c‐Kit*, *Sohlh1*, and *Sohlh2*) (Figure S7J, Supporting Information) in sorted germ cells from *Cwf19l2‐*SKO mice. In addition, flow cytometry analysis revealed that THY1 (an SSC marker)‐positive cells were identified at levels of 2.87% in control testes and 2.89% in *Cwf19l2*‐SKO testes (Figure 5Q), while c‐KIT‐positive cells in *Cwf19l2*‐SKO testes were only half of that in the controls (14.33% *vs* 7.44%, Figure 5T), suggesting that differentiated spermatogonia were reduced as early as PD6 when numerous ASEs occur during the first wave of spermatogenesis.^[^ ^49^ ^]^ These results suggested that absence of CWF19L2 caused impaired spermatogonial differentiation and thus failure of meiosis, while SSCs maintenance and fate determination of spermatogonia undergoing differentiation proceeded normally in *Cwf19l2*‐SKO mice. This effect of *Cwf19l2* knockout suggest a regulatory role in spermatogonial differentiation, most likely through aberrant alternative splicing of spermatogenesis‐related genes.

### 2.6 Deletion of CWF19L2 Disrupts Spliceosome Assembly and Stability

**Figure 6:**
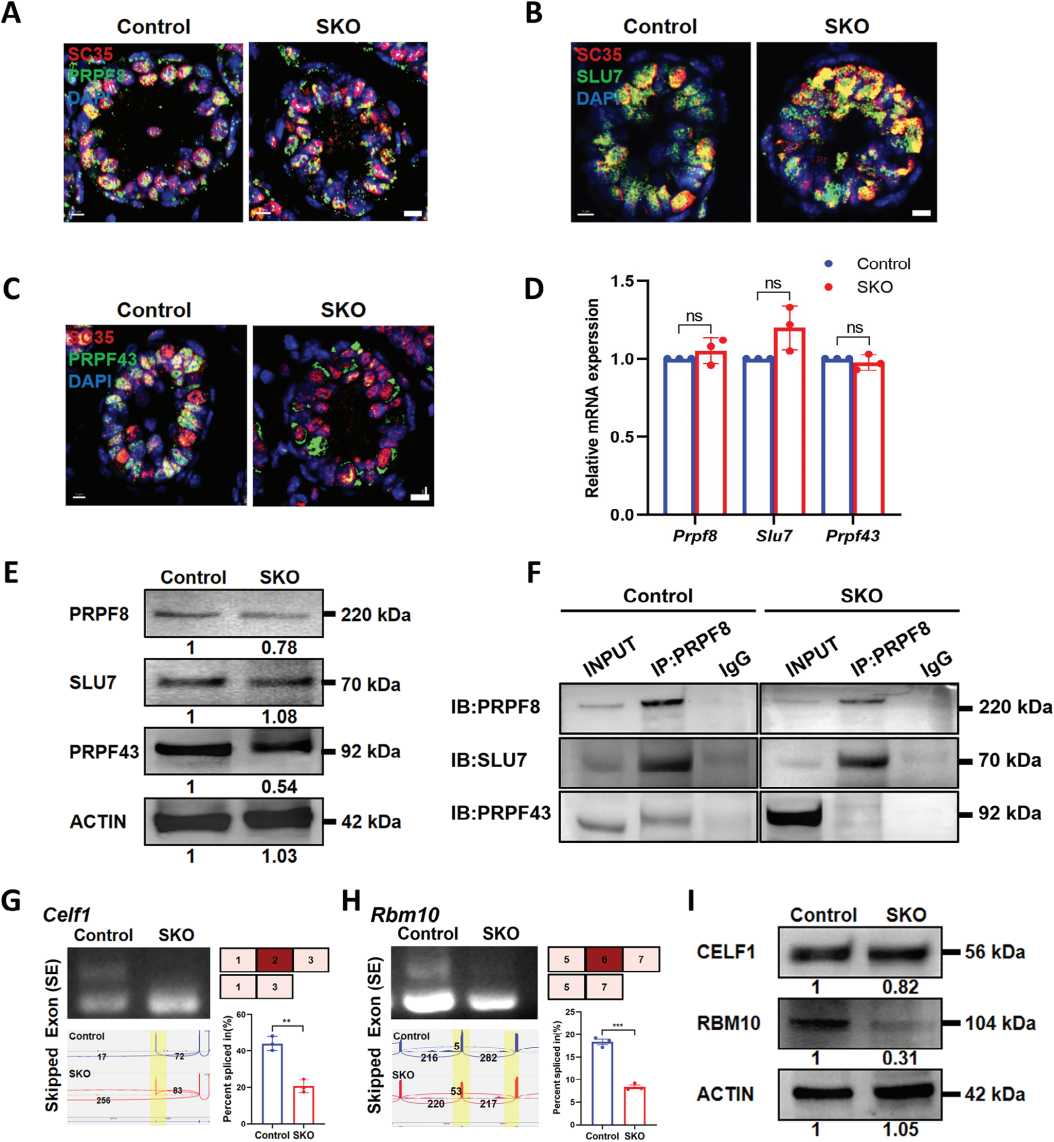
Deletion of CWF19L2 negatively affects spliceosome assembly and stability. A) Co‐immunofluorescence staining of SC35 (red) with the spliceosome skeleton marker PRPF8 (green) in testis sections of PD10 *Cwf19l2*‐SKO and control mice. DNA was stained with DAPI. Scale bars = 5 µm. B) Co‐immunofluorescence staining of SC35 (red) with the P complex marker SLU7 (green) in testis sections of PD10 *Cwf19l2*‐SKO and control mice. DNA was stained with DAPI. Scale bars = 5 µm. C) Co‐immunofluorescence staining of SC35 (red) with the ILS complex marker PRPF43 (green) in testis sections of PD10 *Cwf19l2*‐SKO and control mice. DNA was stained with DAPI. Scale bars = 5 µm. D) QPCR analysis of the mRNA levels of spliceosome‐related genes *Prpf8*, *Slu7*, and *Prpf43* in sorted germ cells from *Cwf19l2*‐SKO and control mice. Data are presented as mean ± SD, n = 3, ns: not significant by two‐tailed Student’ s *t*‐test. E) Immunoblotting analysis of PRPF8, SLU7, and PRPF43 protein in testes of PD10 *Cwf19l2*‐SKO and control mice. ACTIN served as the loading control. F) Co‐IP assays of the interaction between PRPF8, SLU7, and PRPF43 in sorted germ cells from *Cwf19l2*‐SKO and control mice. IgG was used as the negative control. G) Visualization and validation of *Celf1* abnormal ASEs in sorted germ cells from *Cwf19l2*‐SKO and control mice. H) Visualization and validation of *Rbm10* abnormal ASEs in sorted germ cells from *Cwf19l2*‐SKO and control mice. I) Immunoblotting analysis of CELF1 and RBM10 protein in testes of PD10 *Cwf19l2*‐SKO and control mice. ACTIN served as the loading control.

Considering that CWF19L2 interacted with several spliceosome proteins and its knockout led to aberrant alternative splicing of several genes required for splicing, we were therefore sought to define the effects of *Cwf19l2* knockout on the spliceosome. We thus performed co‐immunofluorescence staining of SC35 with PRPF8, SLU7, or PRPF43, and the results indicated that localization of PRPF8 and SLU7 in nuclear speckle was unaffected (**Figure** **6**A,B) while normal PRPF43 signals were almost undetectable in *Cwf19l2*‐SKO mice (Figure 6C). Although the mRNA expression levels of *Prpf8*, *Slu7*, and *Prpf43* did not significantly differ between *Cwf19l2*‐SKO and control mice (Figure 6D), immunoblotting revealed normal SLU7 expression, slightly reduced PRPF8 expression, and moderately reduced PRPF43 expression in *Cwf19l2*‐SKO testes at both PD6 and PD10 (Figure 6E; Figure S8A, Supporting Information). Using sorted germ cells from *Cwf19l2*‐SKO and control mice, co‐IP assays showed PRPF8 specifically co‐precipitated with both PRPF43 and SLU7 in controls, while in *Cwf19l2*‐SKO mice, the interaction between PRPF8 and SLU7 was detectable, but the interaction between PRPF8 and PRPF43 was barely noticeable (Figure 6F). As the lower efficiency of PRPF8 immunoprecipitation in *Cwf19l2*‐SKO testes may be related to the reduced PRPF8 protein levels noted above, which could, in turn, potentially affect PRPF43 binding, we conducted a quantitative analysis to validate our co‐IP results. This analysis indicated that PRPF8 and PRPF43 protein levels decreased by ≈65% and 75% (Figure S8B and C, Supporting Information), respectively, while SLU7 enrichment increased by ≈80% (Figure S8D, Supporting Information), suggesting that SLU7 did not dissociate from the spliceosome in a sufficiently timely manner to prevent stalling. Furthermore, even after normalizing our protein quantification to PRPF8 levels in control samples, i.e., assuming equivalent levels of PRPF8 between WT and *Cwf19l2*‐SKO samples, we still detected 1.5 times higher levels of PRPF43 in controls than in *Cwf19l2*‐SKO mice (Figure S8E, Supporting Information). Therefore, we speculated that CWF19L2 knockout affected the stability and assembly of the spliceosome, which may lead to spliceosome stalling at the P‐ILS transition, likely disrupting the subsequent RNA splicing process.

In addition, we confirmed some genes of alternative mRNA splicing, such as *Celf1* and *Rbm10*, which are essential for recognizing exon‐intron boundary and branchpoint,^[^ ^50^, ^51^ ^]^ and known to play a role in spermatogenesis,^[^ ^52^ ^]^ indeed underwent splicing errors and decreased protein expressions in *Cwf19l2*‐SKO mice (Figure 6G–I, Supporting Information). These results indicate that CWF19L2 is required for the correct assembly and stability of the late‐stage spliceosome.

### 2.7 CWF19L2 Directly Regulates Alternative Splicing and Modulates Other Splicing Factors to Control Gene Splicing Indirectly

**Figure 7:**
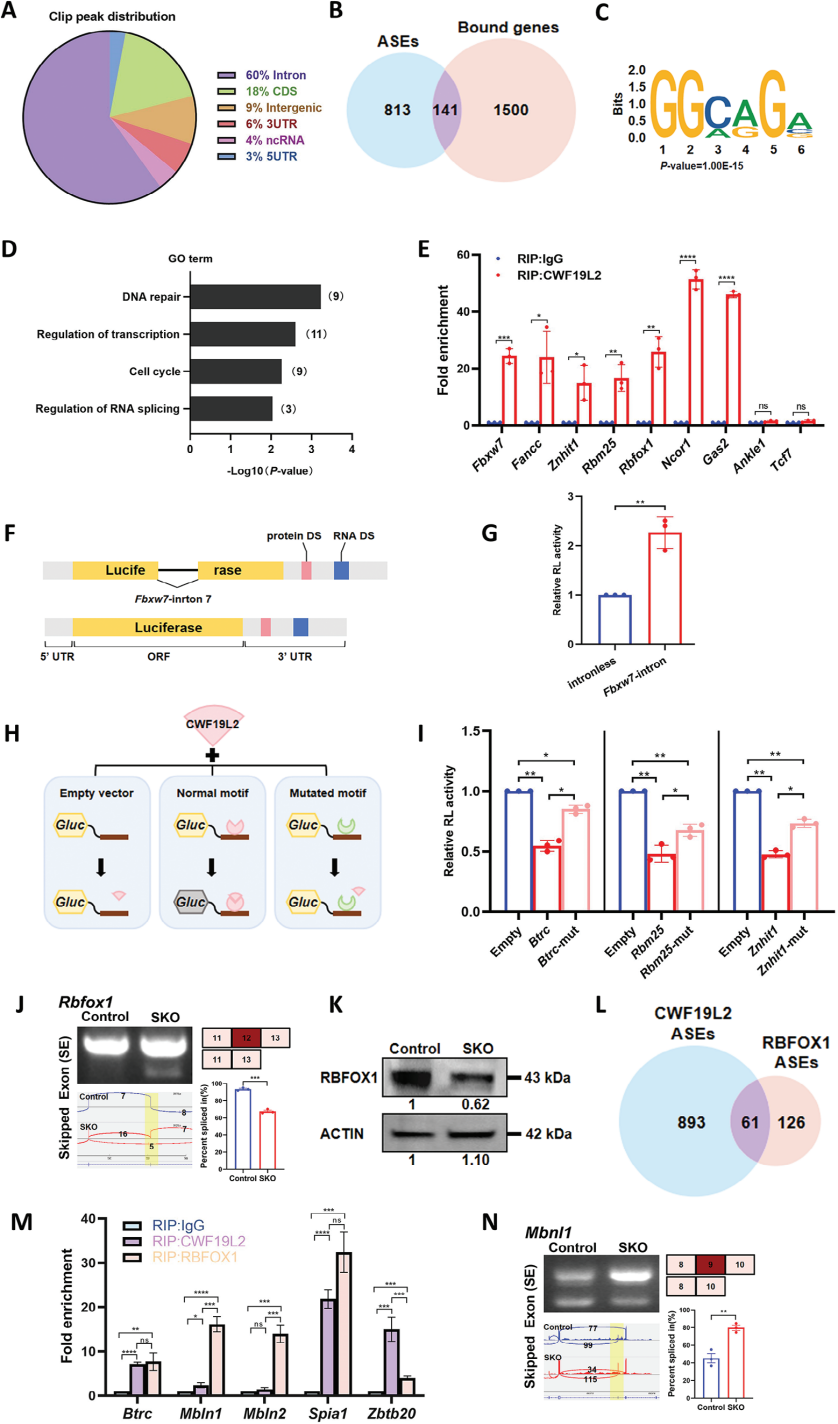
CWF19L2 directly regulates alternative splicing and modulates other splicing factors to control gene splicing indirectly. A) Pie chart showing the distribution of CWF19L2 binding locations in the genome from LACE‐seq in spermatogonia isolated from Cwf19l2‐SKO and control mice. CDS, coding sequence; UTR, untranslated region; ncRNA, non‐coding RNA. B) Venn diagram showing the shared genes between CWF19L2 bound genes and genes with abnormal ASEs. C) HOMER de novo motif analysis of CWF19L2 binding peaks based on the LACE‐seq that shows the identified G‐rich motif. D) GO term enrichment analysis of shared genes between the CWF19L2‐bound genes and genes with abnormal ASEs. E) Histograms showing RIP‐qPCR analysis of some selected mRNAs of the 141 common genes using an anti‐CWF19L2 antibody and control IgG. Data are presented as the mean ± SD, n = 3, ns: not significant, *P < 0.05, **P < 0.01, ***P < 0.001, ****P < 0.0001 by two‐tailed Student’ s t‐test. F) Schematic diagram of the splicing assay performed to assess the splicing of Fbxw7‐intron 7. G) The relative luciferase reporter activity of vectors with or without Fbxw7‐intron 7. H) Schematic diagram of the motif‐recognized luciferase experiment. I) The relative luciferase reporter activity of normal and mutated motif vectors of Btrc, Rbm25 and Znhit1. The empty luciferase vehicle served as the control. J) Visualization and validation of Rbfox1 abnormal ASEs in germ cells from Cwf19l2‐SKO and control mice. K) Immunoblotting analysis of the RBFOX1 protein in testes of PD10 Cwf19l2‐SKO and control mice. ACTIN served as the loading control. L) Venn diagram showing the common genes with ASEs between RNA‐seq data of CWF19L2 and RBFOX1. M) Histograms showing the RIP‐qPCR analysis of five selected mRNAs immunoprecipitated by anti‐CWF19L2 and anti‐RBFOX1 antibodies and control IgG. Data are presented as the mean ± SD, n = 3, ns: not significant, *P < 0.05, **P < 0.01, ***P < 0.001, ****P < 0.0001 by two‐tailed Student’ s t‐test. N) Visualization and validation of Mbnl1 abnormal ASEs in germ cells from Cwf19l2‐SKO and control mice.

To explore the underlying molecular mechanisms through which CWF19L2 regulates pre‐mRNA AS, we performed high‐resolution linear amplification of complementary DNA ends and sequencing (LACE‐seq)^[^ ^53^ ^]^ using FACS‐purified differentiated spermatogonia from *Cwf19l2*‐SKO and control testes. LACE‐seq data robustly confirmed the ability of CWF19L2 to directly bind RNA (**Figure** **7**A), reinforcing the classification as an RNA‐binding protein. In addition, we identified 27542 peaks and 1641 genes that were significantly enriched in the CWF19L2 immunoprecipitants (Figure 7B and Data 5, Supporting Information) (cutoff: fold change >2, *P*‐value < 0.05), and the binding sites preferred the intron regions of the encoded proteins (Figure 7A), aligning with the role of CWF19L2 in ILS complex.^[^ ^11^ ^]^ Motif analysis by the HOMER algorithm indicated that CWF19L2 preferentially recognized the GGMRGV (M = A or C, R = A or G, V = A or C or G) motif, and 57.98% of target sequences contained a GGCAGA motif (Figure 7C).

Comparing the ASEs identified by RNA‐seq and the target genes in the LACE‐seq data, we identified 141 common genes (Figure 7B). Through GO analysis, we found that these AS‐altered genes bound by CWF19L2 were mainly related to DNA repair, regulation of transcription, cell cycle, and RNA splicing regulation (Figure 7D). Using RIP‐qPCR, we confirmed that the common critical genes like *Fbxw7*, *Znhit1*, and *Rbm25* were directly bound by CWF19L2, and we regarded *Ankle1* and *Tcf7* belonging to the non‐common genes to be negative controls (Figure 7E; Figure S9A, Supporting Information), thus highlighting the direct regulatory effect of CWF19L2 at the post‐transcriptional level. Moreover, we found that only 58 upregulated (3.5%) and 34 downregulated (2.1%) genes were bound by CWF19L2 (Figure S9B, Supporting Information), suggesting that CWF19L2 might regulate gene expression by other means than splicing. In order to better understand the regulatory mechanisms involved in the splicing effects of CWF19L2, we compared splicing site strength of CWF19L2‐regulated ASEs with that of the same number of non‐CWF19L2‐regulated ASEs. Our analysis indicated that while both groups showed lower splicing strength at alternative 3′ splice sites relative to constitutive 3′ splice sites (Figure S9C, Supporting Information), splicing strength at alternative 5′ splice sites was exclusively reduced in CWF19L2‐regulated genes (Figure S9D, Supporting Information). Moreover, comparison of splicing strength score ratios indicated that CWF19L2 had a more pronounced impact at 3′ splice sites (Figure S9E, Supporting Information). In light of this analysis, we hypothesized that CWF19L2 was uniquely involved in regulating alternative splicing events in which 3′ splice sites are stronger than 5′ splice sites.

Subsequently, we conducted a splicing reporter assay to verify the impact of CWF19L2 on AS. We integrated the cloned Fbxw7 intron 7 sequence into splicing reporter plasmids (Figure 7F). These plasmids, designed to express luciferase either with or without the Fbxw7 intron 7, were co‐transfected into HEK 293T cells along with a CWF19L2 overexpression vector. Results demonstrated that CWF19L2 significantly enhanced the luciferase activity in the reporter containing *Fbxw7* intron 7 (Figure 7G). At the same time, we performed dual‐luciferase reporter assays to elucidate the regulatory effects of CWF19L2 on specific genes. In these assays, we evaluated the function of CWF19L2 by inserting cloned regions containing CWF19L2‐binding motifs into downstream of the luciferase reporter encoding sequence. We analyzed segments featuring CWF19L2‐binding motifs from *Znhit1* (GGAAGG), *Btrc* (GGAGGA), and *Rbm25* (GGAAGG) (Figure S9F, Supporting Information). Additionally, we created deletion variant control plasmids for each segment examined, such as the “*Znhit1*‐mut” (Figure S9F, Supporting Information). Subsequently, HEK 293T cells were co‐transfected with CWF19L2 overexpression vector and one of the vectors containing these specific segments or an empty vector as a control (Figure 7H). Although there was no discernible change in the reporter mRNA expression levels (Figure S9G, Supporting Information), the luciferase activity in cells containing the CWF19L2‐binding motifs was diminished about by 50% in comparison to the cells with the empty vector control (Figure 7I). Importantly, the reduction in luciferase activity was partially reversed in cells co‐transfected with the deletion variant control vectors for the *Znhit1*, *Btrc*, and *Rbm25* (Figure 7I). These results indicate that CWF19L2 directly interferes with special motifs to modulate splicing.

Approximately 85% of AS‐altered genes were not the direct targets of CWF19L2 (Figure 7B), and we speculated this might be mediated by other splicing factors. Among common genes assigned to RNA splicing regulation, we identified *Rbfox1* as a direct target of CWF19L2 (Figure 7E), We observed the abnormal splicing that skipped exon 12 (Figure 7J), and the decreased RBFOX1 protein in *Cwf19l2*‐SKO mice (Figure 7K). Therefore, we compared the reported RNA‐seq data of *Rbfox1* knockout model^[^ ^54^ ^]^ with our RNA‐seq and obtained 61 shared AS‐altered genes (Figure 7L). GO analysis linked them mainly to RNA processing and apoptotic processes (Figure S9H, Supporting Information). Subsequent RIP‐qPCR confirmed that *Mbln1* and *Mbln2* were bound by RBFOX1, but not CWF19L2, both *Btrc* and *Spia1* were bound by both RBFOX1 and CWF19L2, and *Zbtb20* seemed to be primarily targeted by CWF19L2 (Figure 7M). Relevant ASEs were also verified in *Cwf19l2*‐SKO mice (Figure 7N; Figure S9I–K, Supporting Information). These findings indicated that CWF19L2 can modulate the AS of other splicing factors like RBFOX1 to amplify its effect on splicing regulation.

## 3 Discussion

Spermatogenesis depends on the coordinated control of appropriate gene splicing and expression, thus, it is excellent to study the post‐transcriptional regulation in this intricate process with splicing factors and AS being stage‐specifically modulated.^[^ ^5^, ^55^ ^]^ For instance, SRSF1 and SRSF2 can directly affect AS of *Star8* and other spermatogenesis‐related genes, which have crucial functions in spermatogenesis and male fertility.^[^ ^21^, ^22^ ^]^ However, limited mechanisms are known about how AS functions as a critical regulatory mechanism in spermatogenesis.

In our research, we constructed conditional knockout mouse models to investigate the role of CWF19L2 in spermatogenesis based on its high expression level in testis, especially in germ cells. Our results demonstrated that deletion of CWF19L2 in germ cells caused spermatogenesis failure and complete male infertility, but the knockout of CWF19L2 in Sertoli cells did not affect fertility. In *Cwf19l2*‐SKO mice, while self‐renewal of SSCs and mitosis of undifferentiated spermatogonia proceeded normally, we observed significant apoptosis in differentiated spermatogonia as early as PD6, accelerating to a peak at PD10, and no spermatogonia entering meiosis and sperm were detected eventually. This acceleration of apoptosis may be due to the fact that differentiated spermatogonia have a faster proliferation rate than SSCs^[^ ^56^ ^]^ or to the increased STRA8 expression.^[^ ^57^ ^]^ These findings suggest that CWF19L2 deficiency can lead to the developmental arrest in differentiated spermatogonia and CWF19L2 may also be involved in initiating meiosis.

As a functionally unclear core component of the ILS complex, recruitment of CWF19L2 into the space between PRPF8 and the intron lariat is required for pre‐mRNA splicing. Given the evolutionary conservation, structural resemblance, and similar nuclear localization of CWF19L2, we hypothesize CWF19L2 is functionally conserved in mice. IP‐MS revealed CWF19L2 interacted with several spliceosome proteins, underscoring it could contribute to splicing by spliceosome. In *Cwf19l2*‐SKO mice, the expression of SLU7 remained unchanged, and its increased enrichment through immunoprecipitation using PRPF8‐antibody indicated that it hardly disassembled from the P complex timely. The decreased expression and enrichment of PRPF43 suggested formation of the ILS complex was difficult, meaning that the spliceosome assembly was blocked at P‐ILS transition. The expression and enrichment of PRPF8, a spliceosome structural protein, slightly decreased, which may lead to spliceosome instability. Besides, some interacting proteins of CWF19L2 are associated with spermatogenesis, such as PABPC1 and DDX5.^[^ ^17^, ^37^ ^]^ DDX5 interacts with PLZF to regulate the expression of genes required for germline maintenance and activity of undifferentiated spermatogonia.^[^ ^17^ ^]^ Thus, CWF19L2 is crucial for proper assembly and structural stability of spliceosome to ensure accurate splicing, ultimately guaranteeing successful spermatogenesis.

The RNA‐seq data revealed that, as a splicing factor, depletion of CWF19L2 in spermatogonia affected the splicing of over 850 genes involved in fundamental biological processes such as alternative splicing (*Rbfox1, Celf1*, and *Rbm10*) and spermatogenesis (*Znhit1, Btrc* and *Fbxw7*). These genes play essential roles in mouse spermatogenesis, and their aberrant splicing was verified in sorted germ cells by RT‐PCR. For example, deletion of CWF19L2 caused exon 2 of *Znhit1* to be skipped and the amount of ZNHIT1 to be reduced, which blocks meiotic initiation by arresting male germline cells at the differentiated spermatogonia stage.^[^ ^44^ ^]^ BTRC, which controls the mitosis‐meiosis transition in mouse male germ cells, tended to be expressed as the shortened isoform, skipping exon 3 in *Cwf19l2*‐SKO mice. The *Btrc*‐deficient male germ cells did not enter meiosis but instead underwent apoptosis because of the massive accumulation of DMRT1,^[^ ^45^, ^46^, ^58^ ^]^ whose degradation was required for spermatogonia to exit mitosis and enter meiosis.^[^ ^59^ ^]^ Our findings also detected increased DMRT1, suggesting that its degradation was inhibited, which might be one of the causes of the abnormal spermatogenesis in *Cwf19l2*‐SKO mice. FBXW7, whose intron 7 was retained, is expressed in spermatogonia in a cell cycle‐dependent manner, acting as a negative regulator of SSCs. Deletion of FBXW7 in germ cells suppresses differentiation by upregulation of MYC and CCNE1 and leads to male infertility,^[^ ^40^, ^42^, ^43^ ^]^ which is similar to the phenotype of *Cwf19l2*‐SKO mice in this study. Exon 3‐skipped RBM25 controls the splicing of critical pre‐mRNAs, like the apoptotic regulator *Bcl‐x* and the MYC inhibitor *Bin1*.^[^ ^41^ ^]^ MYC‐mediated apoptosis in spermatogonia following deletion of *Cwf19l2* may be a direct consequence of splicing defects, and this might also explain why apoptosis‐related genes were not predicted to be aberrantly spliced but just differentially expressed. Other genes, including *Celf1*, *Nabp1*, *Tcf7*, and *Gas2*,^[^ ^60^, ^61^, ^62^, ^63^ ^]^ may also be responsible for the aberrant spermatogenesis in *Cwf19l2*‐SKO mice. These abnormalities in splicing, regulated by CWF19L2, might play a role in the growth retardation of the germ cell and male fertility.

Despite depletion of *Cwf19l2* in spermatogonia leading to anomalous alterations in splicing, only 141 genes were directly bound by CWF19L2, as detected by LACE‐seq. CWF19L2 directly regulated AS of *Znhit1* and *Rbm25* through GGAAGG motif and *Btrc* through GGAGGA motif. Knocking out some of these direct targets results in infertile phenotypes similar to those observed in *Cwf19l2*‐SKO mice, reinforcing the critical role of CWF19L2 in spermatogenesis. CWF19L2 directly regulates some splicing factors, such as *Rbfox1*. RIP‐qPCR confirmed that many genes, like *Spia1* and *Btrc*, are directly regulated by both RBFOX1 and CWF19L2, consistent with a previous study showing that most exons are under combinatorial control from different splicing regulator proteins,^[^ ^5^ ^]^ while some genes such as *Mbln1/2* are directly regulated by RBFOX1 but not by CWF19L2. *Mbnl1/2* can repress alternative splicing to balance embryonic stem cell differentiation.^[^ ^64^ ^]^ Therefore, besides direct regulation, CWF19L2 also modulates splicing factors to indirectly control splicing, highlighting the complexity of splicing regulation in spermatogenesis.

Interestingly, beyond splicing alteration, the depletion of *Cwf19l2* in spermatogonia led to gene expression changes. Upregulated genes including transcription factors *(Tnf*, *Cebpa*, *Cebpb*, *Cebpd*) and co‐activators or co‐repressors (*Hes5*, *Hes7*, *Pou2f2*, *Pou3f1*), suggesting CWF19L2 knockout may disrupte the highly dynamic and tightly controlled transcription process. Pre‐mRNAs with abnormal AS may be degraded by nonsense‐mediated mRNA decay(NMD), which is used to monitor the premature termination or truncation of mRNA, resulting in the altered protein expression.^[^ ^47^, ^65^ ^]^ However, only 5.5% of DEGs coincided with ASEs, suggesting that dysregulation of mRNA expression might stem from mechanisms beyond just abnormal AS. Similarly, very few of the DEGs (5.6%) were direct targets of CWF19L2, indicating the potential independent and indirect regulatory roles of CWF19L2 at transcriptional and post‐transcriptional levels.

**Figure 8:**
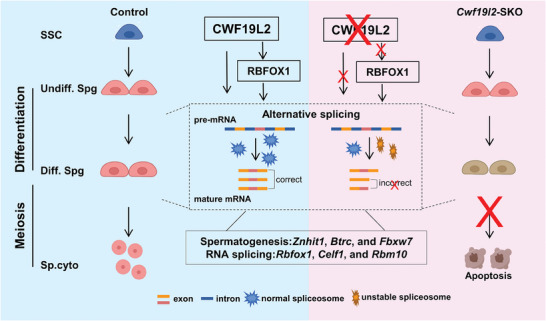
Schematic model of CWF19L2‐mediated AS regulation in the early steps of spermatogenesis. Schematic model showing that CWF19L2 directly binds to target genes to coordinate the proper AS of mRNA transcripts and that CWF19L2 controls key splicing factors like RBFOX1 to indirectly amplify its role in splicing regulation during spermatogenesis.

In conclusion, our research underscores that CWF19L2 is an essential splicing regulator, illustrating its direct and indirect influence on the AS of critical genes required for alternative splicing (*Rbfox1, Celf1*, and *Rbm10*) and spermatogenesis (*Znhit1, Btrc*, and *Fbxw7*) in germ cells to ensure successful spermatogenesis (**Figure** **8**). This research significantly advances our understanding of the AS machinery underpinning spermatogenesis and offers new perspectives on the genetic basis of male fertility.

## 4 Experimental Section

### Mice and Genotyping

*Cwf19l2^flox/+^* mice were produced through embryonic stem cells (ESCs) targeting and blastocyst injection in a C57BL/6J genetic background from Cyagen Biosciences. ESCs incorporated two loxP sites flanked in exon 6. *Stra8‐GFPCre* mice line in the C57BL/6 J background were a generous gift from Prof. Ming‐han Tong at the Center for Excellence in Molecular Cell Science, Chinese Academy of Sciences.^[^ ^66^, ^67^ ^]^ Initially, 8‐week‐old *Stra8‐GFPCre* males were crossed with 6‐week‐old *Cwf19l2^flox/flox^* females to generate the *Cwf19l2^flox/+^*; *Stra8‐GFPCre* mice. Subsequently, the 8‐week‐old *Cwf19l2^flox/+^*; *Stra8‐GFPCre* mice were bred with *Cwf19l2^flox/+^ or Cwf19l2^flox/flox^* mice to obtain *Cwf19l2^flox/flox^*; *Stra8‐GFPCre* (designated as *Cwf19l2*‐SKO or SKO) mice. All mice were maintained in a controlled lighting regime (12 h light; 12 h darkness) at 21–22 °C with unlimited access to water and food. Genotyping was performed by PCR on genomic DNA extracted from PD5 mouse tails. The PCR primer sequences were shown in Data 6 (Supporting Information). All experimental protocols adhered to the ethical guidelines set by the regional ethics committee of Shandong University.

### Fertility Test

Fertility in control and *Cwf19l2‐*SKO mice after 8 weeks old was evaluated. Each *Cwf19l2‐*SKO male was caged with two 8–12 week‐old WT C57BL/6J female mice. Female mice were checked daily for vaginal plugs, and if detected, were separated into single cage for pregnancy recording. The fertility test continued for at least 6 months.

### Histological Analysis

After euthanasia, mouse testes and epididymides were fixed in Bouin's Fixative Solution (Scientific phygene, PH0976) at 4 °C overnight. Following dehydration, tissues were embedded in paraffin, sectioned at 5 µm thickness, and mounted on glass slides. After deparaffinization, the slides were stained with hematoxylin using a standard protocol. The hematoxylin‐stained slides were imaged with a fluorescence microscope (BX53, Olympus), and images were processed using Image J.

### Immunofluorescence

After euthanasia, mouse testes and epididymides were fixed in 4% paraformaldehyde (Solarbio, P1110) at 4 °C overnight. Following dehydration, tissues were embedded in paraffin, sectioned at 5 µm thickness, and mounted on glass slides. After deparaffinization, the slides were boiled in Citrate Antigen Retrieval Solution (Beyotime, P0081) for 15 min in boiling water and then cooled to room temperature. Sections were treated with phosphate‐buffered saline (PBS) containing 0.1% Triton X‐100 for 15 min, followed by washing three times in PBS (pH 7.4). Then slides were blocked using 5% bovine serum albumin for 1 h at room temperature, and incubated with primary antibodies at 4 °C overnight. Washing with PBS three times, secondary antibodies were added and incubated for 1 h at room temperature. Washing with PBS three times again, the slides were mounted using DAPI aqueous, fluoroshield (Abcam, ab104139). Immunostained slides were imaged by confocal microscopy (Andor Dragonfly spinning disc confocal microscope driven by Fusion Software). The details of the primary and secondary antibodies used in this study were detailed in Data 7 (Supporting Information).

### Tunel Analyses

One Step TUNEL Apoptosis Assay Kit (KeyGEN BioTECH, KGA7072) was utilized following the manufacturer's instructions on testes sections.

### Immunoblotting

Tissues were collected to prepare protein extracts from C57BL/6 mice and lysed in NP‐40 Lysis Buffer (Beyotime, P0013F) with protease inhibitor (Roche, 04693132001). After homogenization, the cell extracts were chilled on ice stand for 20 min, and centrifuged at 4 °C at 13 000 ×g for 20 min. The supernatant was denatured with 5×SDS loading buffer (Beyotime, P0015L) and heated to 95 °C for 5 min. Equal total proteins were separated on a 10% SDS–PAGE gel (Invitrogen, NP0315) and transferred to PVDF membranes (Millipore, ISEQ00010). Membranes were blocked with 5% nonfat milk for 1 h at room temperature and incubated with primary antibodies overnight at 4 °C. After washing three times with TBST, membranes were incubated with the secondary antibodies for 1 h at room temperature. The signals were detected by Luminol/enhancer solution and Peroxide solution (ClarityTM Western ECL Substrate, BioRad). The details of the primary and secondary antibodies used in this study were detailed in Data 7 (Supporting Information).

### Immunoprecipitation

The supernatant obtained by method in immunblotting was incubated with antibodies and spined at 4 °C overnight. The pre‐cleaned magnetic protein A/G beads were added and the mixture spined at 4 °C for 2 h. The beads were washed with NP‐40 Lysis Buffer (Beyotime, P0013F) with protease inhibitor (Roche, 0 469 313 2001) three times and boiled in 2×SDS loading buffer (Beyotime, P0015L) for immunoblotting analyses.

### Fluorescence‐Activated Cell Sorting of Spermatogonia

After removing the tunica albuginea, the testes were placed in 5 mL PBS containing collagenase type I (120 U mL^−1^) and spined for 10 min at 35 °C. Then testes were digested in 5 mL of 0.25% trypsin plus 0.1 mL of deoxyribonuclease I (5 mg mL^−1^) at 35 °C for 8 min and terminated by adding of 0.5 mL fetal bovine serum. The suspension was filtered through a 40 µm honeycomb filter and centrifuged at 4 °C at 500 ×g for 5 min. Removing the supernatant, cells were resuspended in 1 mL Dulbecco's modified Eagle medium (DMEM) with Hoechst 33 342, PE anti‐mouse CD117 (c‐Kit) antibody (Biolegend,135 105) or APC/Cyanine7 anti‐mouse Thy1 antibody (Biolegend,105 328), and 5 µL DNase I. The cell suspension was stirred at 34 °C for 20 min, centrifuged at 500 ×g for 5 min at 4 °C and resuspended in PBS at a concentration of 10^5^ mL for sorting. Fluorescently labeled cells were collected according to the sorting channel into 1.5 mL LoBind microcentrifuge tubes (Eppendorf, 02 243 1021) containing 0.5 mL PBS for subsequent analysis.

### Enrichment of Spermatogenic Cells and Sertoli Cells

Testes were digested into single cells according to the above. Then suspension was suspended in 10 mL of DMEM medium with 10% FBS and 1% penicillin‐streptomycin, and seeded in a 10 cm culture dish. After incubating at 37 °C for 3 h, the floating and weakly adhering cells were harvested as the fraction of spermatogenic cells, while the cells attached to the bottom of the dish were collected as the fraction of somatic cells. The efficiency of the separation was determined using immunoblotting analysis.

### RNA Extraction, qPCR, RT‐PCR, RNA‐seq and SMART‐seq

Total RNA was extracted from whole testes or sorted cells using FastPure Cell/Tissue Total RNA Isolation Kit V2 (Vazyme, RC112‐01) following the manufacturer's instructions. For qPCR, isolated total RNA was reverse‐transcribed into first‐strand cDNA using the HiScript III RT SuperMix for qPCR (+gDNA wiper) (Vazyme, R323‐01). qPCR was performed with the SYBR Green Premix Pro Taq HS qPCR Kit (AG, 11 701) on LightCycler@96 Real‐Time PCR system (Roche) according to manufacturer's instructions. *Gapdh* (LOC107788267) was employed as an internal control and each experiment conducted in triplicate to ensure technical reliability. Finally, relative gene expression were calculated using the 2^−∆∆Ct^ method, which was subsequently converted to ploidy changes for graphical representation. For RT‐PCR, isolated total RNA was reverse‐transcribed into first‐strand cDNA using the HiScript III 1st Strand cDNA Synthesis Kit (+gDNA wiper) (Vazyme, R312‐02). cDNA and specialized primers underwent corresponding PCR cycles. The products were separated by agarose gel and quantified by Image J software. Splicing ratios were represented as PSI (Percent Spliced In) value, representing the percentage of a gene's mRNA transcripts that included a specific exon or splice site. The primers for RT‐PCR and qPCR assay are listed in Supplementary Data 6. For RNA‐seq, isolated total RNA was converted into cDNA for RNA sequencing using Illumina Truseq RNA Sample Preparation Kit, followed by rRNA removal and sequenced on an Illumina HiSeq 2500 using 2 × 150 nt sequencing. For SMART‐seq, the Discover‐sc WTA Kit V2 (Vazyme, N711) facilitated cell lysis, mRNA reverse transcription, and cDNA amplification. Subsequent cDNA fragmentation and adapter ligation steps were performed using the TruePrep Flexible DNA Library Prep Kit for Illumina kit (Vazyme, TD504). All experimental procedures were conducted strictly following the manufacturer's instructions. The Smart‐seq2 library sequencing was carried out by Novogene on the Illumina platforms with 150 bp paired‐end reads.

### LACE‐seq

Crosslinking immunoprecipitation coupled with high‐throughput sequencing of FACS‐purified spermatocyte populations was performed according to published study.^[^ ^53^ ^]^ Immunoprecipitation was conducted using the Rab‐CWF19L2 antibody, applying 3 µg per test sample, while rabbit IgG served as the negative control. Spermatogonia cells, sorted as previously described, were collected in 1.5 ml LoBind microcentrifuge tubes, each containing 10 µl PBS. The cells underwent UV‐C light irradiation twice on ice at 400 mJ, with a 1–2 min pause between irradiations. Following the methodology in reference,^[^ ^50^ ^]^ the initial stages of LACE‐seq, including RNA immunoprecipitation and fragmentation, RNA dephosphorylation and 3′ linker ligation, RT on beads, and first‐strand cDNA capture by streptavidin beads, were meticulously executed. Substituting the poly(A) tailing, a 3′ cDNA linker was added. The streptavidin beads with first‐strand cDNA were resuspended in 20 µl ligation mixture (10 µl 50% PEG8000, 4.5 µl water, 2 µl 10× ligation buffer, 2 µl ATP (10 µM, NEB, P0756S), 1 µl T4 RNA ligase 1, truncated (NEB, M0204S), 0.5 µl 3′ cDNA linker (1 µM, /5pA/CTCGTATGCCGTCTTCTGCTTG/3 NH2/, where 5pA denotes 5′ phosphorylated adenosine triphosphate, and 3 indicates 3′‐amino, synthesized and HPLC‐purified by Sangon) and transferred to a new PCR tube for a 24 h incubation at 25 °C in a ThermoMixer C with intermittent vortexing for 15 s at 1000 rpm every 3 min. After that, to create the dsDNA template for in vitro transcription (IVT), the DNA was pre‐amplified by first‐PCR with the following mixture: 12.5 µl cDNA, 0.5 µl primer A (GATCACTAATACGACTCACTATAGG, 10 µM; Sangon), 0.5 µl P7 primer (CAAGCAGAAGACGGCATACGAGAT, 10 µM; Sangon) and 12.5 µl 2× KAPA HiFi HotStart ReadyMix (KAPA Biosystems, KK2601). The PCR program was set as follows: 98 °C for 3 min; 98 °C for 15 s, 60 °C for 20 s, and 72 °C for 30 s (14–18 cycles); 72 °C for 5 min, then hold at 12 °C for a long time. The PCR tube was then placed on a magnetic rack for 2 min, and the supernatant was transferred to a new LoBind tube and purified by 46.8 µl Ampure XP beads (1.8:1 ratio; Beckman Coulter, A63881) according to the manufacturer's instructions. 13 µl Water was added to the LoBind tubes to elute PCR products and then transferred to a new PCR tube. The subsequent IVT, RNA purification and RT were conducted sequentially. PCR barcoding was performed with 20 µl cDNA, 1 µl P7 primer, 1 µl P5 index primer (AATGATACGGCGACCACCGAGATCTACACNNNNNACACT, 10 µM; Sangon) and 22 µl 2×KAPA HiFi HotStart ReadyMix. And the PCR program was set as follows: 94 °C for 3 min; 94 °C for 15 s, 62 °C for 30 s, and 72 °C for 30 s (8–12 cycles); 72 °C for 10 min, then hold at 12 °C. PCR products between 130 and 300 bp were extracted by Agarose gel electrophoresis with a 2% agarose gel and then purified using a gel extraction kit (Qiagen, 28 604). The LACE‐seq library was single‐end sequenced using Illumina HiSeq 2500 at Novogene. LACE‐seq data mapping and identification of peak, cluster, and motif were performed following study.^[^ ^53^ ^]^

### RNA‐Binding Protein Immunoprecipitation

RNA‐protein complex was isolated according to the manufacturer's instructions (Geneseed, P0101). Briefly, Tissues were homogenized in immunoprecipitation lysis buffer with RNasin and protease inhibitor and then divided into two samples for anti‐CWF19L2 or anti‐RBFOX1 and anti‐IgG (serving as the negative control). Protein A/G Magnetic Beads facilitated the capture of targets at 4 °C overnight.The RNA bound to the immunoprecipitated protein was then purified and subjected to qPCR analysis for further evaluation

### Dual‐Luciferase Reporter Assay

Dual‐luciferase reporters assay utilized the Secrete‐Pair Dual Luminescence Assay Kit (GeneCopoeia, LF031). The CWF19L2‐bound fragments of targets and their deletion variant, see as Figure S6C (Supporting Information), were integrated into the pEZX‐GA02 vector at MCS2 sites. *Fbxw7* intron 7 sequence was cloned into CMV‐LUC2CP/intron/ARE plasmids (Addgene). HEK 293T cells were cultured in 12‐well plates and co‐transfected with 500 ng of reporter and CWF19L2 overexpression plasmids using HP DNA Transfection reagent (Roche, 06366546001). 48 h later, cells were collected, and luciferase activities were assessed according to the manufacturer's protocols by Microplate luminometer (Berthold, LB960).

### Statistical Analysis

Quantitative experiments were based on at least three independent biological samples with the data are presented as means ± standard deviation (SD). Statistical analyses were performed using GraphPad Prism 8.0 software (GraphPad, San Diego, CA, USA), employing a two‐tailed Student’ s *t*‐test for pairwise comparisons. The data were considered significant when the *P*‐value was less than 0.05.

## Conflict of Interest

The authors declare no conflict of interest.

## Author Contributions

S.W. and Y.C. contributed equally to this work. S.W. was involved in conceptualization, the majority of experimentation, data collection, analysis, writing original draft, and review and editing. Y.C. performed LACE‐seq experiments. T.L. analyzed LACE‐seq data. Y.W., Z.B. and R.W. performed part of genotyping. J.Q. provided antibodies and advice. Z.W. and Y.L. provided guidance. Z.L., W.‐Y.C., X.C. and G.L. contributed to project administration. Z.‐J.C., T.H., and H.L. were responsible for supervision, project administration, resources, and funding acquisition. All authors have read and approved the final version of the manuscript.

## Supporting information

Supporting Information

Supporting Information

## Data Availability

The data that support the findings of this study are available in the supplementary material of this article.

## References

[1] M. D. Griswold , Physiol. Rev. 2016, 96, 1. 26537427 10.1152/physrev.00013.2015 PMC4698398

[2] D. G. de Rooij , Development 2017, 144, 3022. 28851723 10.1242/dev.146571

[3] H. B. Song , L. Wang , D. K. Chen , F. E. Li , Int J Biol Sci 2020, 16, 38. 31892844 10.7150/ijbs.34422 PMC6930371

[4] D. G. de Rooij , Reproduction 2001, 121, 347. 11226060 10.1530/rep.0.1210347

[5] H. Gan , T. Cai , X. Lin , Y. Wu , X. Wang , F. Yang , C. Han , Mol. Cell. Proteomics 2013, 12, 1144. 23325766 10.1074/mcp.M112.020123 PMC3650327

[6] R. Schmid , S. N. Grellscheid , I. Ehrmann , C. Dalgliesh , M. Danilenko , M. P. Paronetto , S. Pedrotti , D. Grellscheid , R. J. Dixon , C. Sette , I. C. Eperon , D. J. Elliott , Nucleic Acids Res. 2013, 41, 10170. 24038356 10.1093/nar/gkt811 PMC3905889

[7] A. R. Grosso , A. Q. Gomes , N. L. Barbosa‐Morais , S. Caldeira , N. P. Thorne , G. Grech , M. von Lindern , M. Carmo‐Fonseca , Nucleic Acids Res. 2008, 36, 4823. 18653532 10.1093/nar/gkn463 PMC2528195

[8] B. Matos , S. J. Publicover , L. F. C. Castro , P. J. Esteves , M. Fardilha , Open Biol 2021, 11, 200322. 34062096 10.1098/rsob.200322 PMC8169208

[9] J. M. D. Legrand , R. M. Hobbs , Seminars in Cell & Developmental Biology 2018, 79, 80. 29024760 10.1016/j.semcdb.2017.10.006

[10] M. C. Wahl , C. L. Will , R. Luhrmann , Cell 2009, 136, 701. 19239890 10.1016/j.cell.2009.02.009

[11] X. F. Zhang , X. C. Zhan , C. Y. Yan , W. Y. Zhang , D. L. Liu , J. L. Lei , Y. G. Shi , Cell Res. 2019, 29, 274.30728453

[12] R. X. Wan , R. Bai , X. C. Zhan , Y. G. Shi , Annu. Rev. Biochem. 2020, 89, 333. 31815536 10.1146/annurev-biochem-013118-111024

[13] C. Y. Yan , R. X. Wan , Y. G. Shi , Csh. Perspect. Biol. 2019, 11. 10.1101/cshperspect.a032409 PMC6314064 30602541

[14] X. C. Zhan , C. Y. Yan , X. F. Zhang , J. L. Lei , Y. G. Shi , Cell Res. 2018, 28, 1129. 30315277 10.1038/s41422-018-0094-7 PMC6274647

[15] M. P. Paronetto , V. Messina , E. Bianchi , M. Barchi , G. Vogel , C. Moretti , F. Palombi , M. Stefanini , R. Geremia , S. Richard , C. Sette , J. Cell Biol. 2009, 185, 235. 19380878 10.1083/jcb.200811138 PMC2700383

[16] W. Liu , F. Wang , Q. Xu , J. Shi , X. Zhang , X. Lu , Z. A. Zhao , Z. Gao , H. Ma , E. Duan , F. Gao , S. Gao , Z. Yi , L. Li , Nat. Commun. 2017, 8, 14182. 28128212 10.1038/ncomms14182 PMC5290162

[17] J. M. D. Legrand , A. L. Chan , H. M. La , F. J. Rossello , M. L. Anko , F. V. Fuller‐Pace , R. M. Hobbs , Nat. Commun. 2019, 10, 2278. 31123254 10.1038/s41467-019-09972-7 PMC6533336

[18] C. Naro , L. Pellegrini , A. Jolly , D. Farini , E. Cesari , P. Bielli , P. de la Grange , C. Sette , Cell Rep. 2019, 26, 2929. 30865884 10.1016/j.celrep.2019.02.058

[19] S. Feng , J. Li , H. Wen , K. Liu , Y. Gui , Y. Wen , X. Wang , S. Yuan , Nat. Commun. 2022, 13, 3588. 35739118 10.1038/s41467-022-31364-7 PMC9226075

[20] W. Liu , X. Lu , Z. H. Zhao , R. Su , Q. L. Li , Y. Xue , Z. Gao , S. S. Sun , W. L. Lei , L. Li , G. An , H. Liu , Z. Han , Y. C. Ouyang , Y. Hou , Z. B. Wang , Q. Y. Sun , J. Liu , Elife 2022, 11, e78211.36355419

[21] W. L. Lei , Z. Du , T. G. Meng , R. Su , Y. Y. Li , W. Liu , S. M. Sun , M. Y. Liu , Y. Hou , C. H. Zhang , Y. Gui , H. Schatten , Z. Han , C. Liu , F. Sun , Z. B. Wang , W. P. Qian , Q. Y. Sun , BMC Biol. 2023, 21, 231. 37867192 10.1186/s12915-023-01736-6 PMC10591377

[22] W. L. Lei , Y. Y. Li , Z. Du , R. Su , T. G. Meng , Y. Ning , G. Hou , H. Schatten , Z. B. Wang , Z. Han , F. Sun , W. P. Qian , C. Liu , Q. Y. Sun , Int J Biol Sci 2023, 19, 4883. 37781512 10.7150/ijbs.83474 PMC10539708

[23] Y. Lv , G. Lu , Y. Cai , R. Su , L. Liang , X. Wang , W. Mu , X. He , T. Huang , J. Ma , Y. Zhao , Z. J. Chen , Y. Xue , H. Liu , W. Y. Chan , Protein Cell 2023, 14, 51. 36726756 10.1093/procel/pwac040 PMC9871953

[24] J. Qin , T. Huang , Z. Wang , X. Zhang , J. Wang , Q. Dang , D. Cui , X. Wang , Y. Zhai , L. Zhao , G. Lu , C. Shao , S. Li , H. Liu , Z. Liu , Cell Death Differ. 2023, 30, 184. 36114296 10.1038/s41418-022-01057-1 PMC9883385

[25] J. Mata , R. Lyne , G. Burns , J. Bahler , Nat. Genet. 2002, 32, 143. 12161753 10.1038/ng951

[26] S. H. Nordgard , F. E. Johansen , G. I. Alnaes , E. Bucher , A. C. Syvanen , B. Naume , A. L. Borresen‐Dale , V. N. Kristensen , Genes Chromosomes Cancer 2008, 47, 680. 18398821 10.1002/gcc.20569

[27] S. M. Garrey , A. Katolik , M. Prekeris , X. Li , K. York , S. Bernards , S. Fields , R. Zhao , M. J. Damha , J. R. Hesselberth , RNA 2014, 20, 1337. 24919400 10.1261/rna.044602.114 PMC4105757

[28] M. D. Ohi , A. J. Link , L. Ren , J. L. Jennings , W. H. McDonald , K. L. Gould , Mol. Cell. Biol. 2002, 22, 2011. 11884590 10.1128/MCB.22.7.2011-2024.2002 PMC133674

[29] L. Casalino , G. Palermo , A. Spinello , U. Rothlisberger , A. Magistrato , Proc, Natl. Acad. Sci. U S A 2018, 115, 6584. 29891649 10.1073/pnas.1802963115 PMC6042132

[30] S. J. Hill , T. Rolland , G. Adelmant , X. Xia , M. S. Owen , A. Dricot , T. I. Zack , N. Sahni , Y. Jacob , T. Hao , K. M. McKinney , A. P. Clark , D. Reyon , S. Q. Tsai , J. K. Joung , R. Beroukhim , J. A. Marto , M. Vidal , S. Gaudet , D. E. Hill , D. M. Livingston , Genes Dev. 2014, 28, 1957. 25184681 10.1101/gad.241620.114 PMC4197947

[31] S. Malik , H. Saito , M. Takaoka , Y. Miki , A. Nakanishi , Cell Cycle 2016, 15, 2145. 27433848 10.1080/15384101.2016.1195531 PMC4993541

[32] Q. Shen , L. Teng , Y. Wang , L. Guo , F. Xu , H. Huang , W. Xie , Q. Zhou , Y. Chen , J. Wang , Y. Mao , J. Chen , H. Jiang , Front Immunol 2022, 13, 962986. 36159820 10.3389/fimmu.2022.962986 PMC9495259

[33] I. Papatheodorou , P. Moreno , J. Manning , A. M.‐P. Fuentes , N. George , S. Fexova , N. A. Fonseca , A. Füllgrabe , M. Green , N. Huang , L. Huerta , H. Iqbal , M. Jianu , S. Mohammed , L. Zhao , A. F. Jarnuczak , S. Jupp , J. Marioni , K. Meyer , R. Petryszak , C. A. Prada Medina , C. Talavera‐López , S. Teichmann , J. A. Vizcaino , A. Brazma , Nucleic Acids Res. 2019, 48, D77. 10.1093/nar/gkz947 PMC7145605 31665515

[34] M. Lizio , J. Harshbarger , H. Shimoji , J. Severin , T. Kasukawa , S. Sahin , I. Abugessaisa , S. Fukuda , F. Hori , S. Ishikawa‐Kato , C. J. Mungall , E. Arner , J. K. Baillie , N. Bertin , H. Bono , M. de Hoon , A. D. Diehl , E. Dimont , T. C. Freeman , K. Fujieda , W. Hide , R. Kaliyaperumal , T. Katayama , T. Lassmann , T. F. Meehan , K. Nishikata , H. Ono , M. Rehli , A. Sandelin , E. A. Schultes , et al., Genome Biol. 2015, 16, 22. 25723102 10.1186/s13059-014-0560-6 PMC4310165

[35] I. Abugessaisa , J. A. Ramilowski , M. Lizio , J. Severin , A. Hasegawa , J. Harshbarger , A. Kondo , S. Noguchi , C. W. Yip , J. L.i C. Ooi , M. Tagami , F. Hori , S. Agrawal , C. C. Hon , M. Cardon , S. Ikeda , H. Ono , H. Bono , M. Kato , K. Hashimoto , A. Bonetti , M. Kato , N. Kobayashi , J. Shin , M. de Hoon , Y. Hayashizaki , P. Carninci , H. Kawaji , T. Kasukawa , Nucleic Acids Res. 2020, 49, D892. 10.1093/nar/gkaa1054 PMC7779024 33211864

[36] Y. Peng , J. Yuan , Z. Zhang , X. Chang , J. Biol. Chem. 2017, 292, 12285. 28611064 10.1074/jbc.M117.794834 PMC5519376

[37] Y. Y. Wu , K. B. Xu , H. Y. Qi , Biol. Reprod. 2018, 99, 773. 29701755 10.1093/biolre/ioy100

[38] Z. Lin , P. J. Hsu , X. D. Xing , J. H. Fang , Z. K. Lu , Q. Zou , K. J. Zhang , X. Zhang , Y. C. Zhou , T. Zhang , Y. C. Zhang , W. L. Song , G. F. Jia , X. R. Yang , C. He , M. H. Tong , Cell Res. 2017, 27, 1216. 28914256 10.1038/cr.2017.117 PMC5630681

[39] C. Lecureuil , I. Fontaine , P. Crepieux , F. Guillou , Genesis 2002, 33, 114. 12124943 10.1002/gene.10100

[40] M. Kanatsu‐Shinohara , I. Onoyama , K. I. Nakayama , T. Shinohara , Proc. Natl. Acad. Sci. U S A 2014, 111, 8826. 24879440 10.1073/pnas.1401837111 PMC4066470

[41] Y. Ge , M. B. Schuster , S. Pundhir , N. Rapin , F. O. Bagger , N. Sidiropoulos , N. Hashem , B. T. Porse , Nat. Commun. 2019, 10, 172. 30635567 10.1038/s41467-018-08076-y PMC6329799

[42] H. Zhang , F. Chen , H. Dong , M. Xie , H. Zhang , Y. Chen , H. Liu , X. Bai , X. Li , Z. Chen , Biol. Reprod. 2020, 102, 963. 31883011 10.1093/biolre/ioz230

[43] D. Zhou , X. Wang , Z. Liu , Z. Huang , H. Nie , W. Zhu , Y. Tan , L. Fan , Tissue Cell 2020, 62, 101315. 32433022 10.1016/j.tice.2019.101315

[44] S. Sun , Y. Jiang , Q. Zhang , H. Pan , X. Li , L. Yang , M. Huang , W. Wei , X. Wang , M. Qiu , L. Cao , H. He , M. Yu , H. Liu , B. Zhao , N. Jiang , R. Li , X. Lin , Dev. Cell 2022, 57, 901. 35413238 10.1016/j.devcel.2022.03.006

[45] D. Guardavaccaro , D. Frescas , N. V. Dorrello , A. Peschiaroli , A. S. Multani , T. Cardozo , A. Lasorella , A. Iavarone , S. Chang , E. Hernando , M. Pagano , Nature 2008, 452, 365. 18354482 10.1038/nature06641 PMC2707768

[46] A. Morohoshi , T. Nakagawa , S. Nakano , Y. Nagasawa , K. Nakayama , Developmental Bio. 2019, 445, 178. 10.1016/j.ydbio.2018.10.023 30391586

[47] F. Supek , B. Lehner , R. G. H. Lindeboom , Trends Genet. 2021, 37, 657. 33277042 10.1016/j.tig.2020.11.002

[48] J. F. García‐Moreno , L. Romão , Int. J. Mol. Sci. 2020, 21. 10.3390/ijms21249424 PMC7764535 33321981

[49] H. Song , L. Wang , D. Chen , F. Li , Int J Biol Sci 2020, 16, 38. 31892844 10.7150/ijbs.34422 PMC6930371

[50] A. Damianov , C. H. Lin , J. Huang , L. Zhou , Y. Jami‐Alahmadi , J. Wohlschlegel , D. L. Black , Biorxiv : The Preprint Server for Biology 2023, 9424.

[51] H. Xia , D. Chen , Q. Wu , G. Wu , Y. Zhou , Y. Zhang , L. Zhang , Biochim. Biophys. Acta Gene Regul. Mech. 2017, 1860, 911. 28733224 10.1016/j.bbagrm.2017.07.004

[52] A. Dev , K. Nayernia , M. Meins , I. Adham , F. Lacone , W. Engel , Mol. Reproduc. Develop. 2007, 74, 1456. 10.1002/mrd.20742 17393433

[53] R. Su , L. H. Fan , C. Cao , L. Wang , Z. Du , Z. Cai , Y. C. Ouyang , Y. Wang , Q. Zhou , L. Wu , N. Zhang , X. Zhu , W. L. Lei , H. Zhao , Y. Tian , S. He , C. C. L. Wong , Q. Y. Sun , Y. Xue , Nat. Cell Biol. 2021, 23, 664. 34108658 10.1038/s41556-021-00696-9

[54] S. Pedrotti , J. Giudice , A. Dagnino‐Acosta , M. Knoblauch , R. K. Singh , A. Hanna , Q. Mo , J. Hicks , S. Hamilton , T. A. Cooper , Hum. Mol. Genet. 2015, 24, 2360. 25575511 10.1093/hmg/ddv003 PMC4380076

[55] H. White‐Cooper , I. Davidson , Cold Spring Harb. Perspect. Biol. 2011, 3, a002626. 21555408 10.1101/cshperspect.a002626 PMC3119912

[56] D. G. De Rooij , L. D. Russell , J. Androl. 2000, 21, 776. 11105904

[57] P. I. Sadate‐Ngatchou , C. J. Payne , A. T. Dearth , R. E. Braun , Genesis 2008, 46, 738. 18850594 10.1002/dvg.20437 PMC2837914

[58] T. Nakagawa , T. Zhang , R. Kushi , S. Nakano , T. Endo , M. Nakagawa , N. Yanagihara , D. Zarkower , K. Nakayama , Development 2017, 144, 4137. 28982686 10.1242/dev.158485 PMC5719248

[59] T. Zhang , D. Zarkower , Stem Cell Res. 2017, 24, 195. 28774758 10.1016/j.scr.2017.07.026 PMC5634931

[60] C. Kress , C. Gautier‐Courteille , H. B. Osborne , C. Babinet , L. Paillard , Mol. Cell. Biol. 2007, 27, 1146. 17130239 10.1128/MCB.01009-06 PMC1800704

[61] D. Hrckulak , M. Kolar , H. Strnad , V. Korinek , Cancers (Basel) 2016, 8. 10.3390/cancers8070070 PMC4963812 27447672

[62] J. P. York , Y. A. Ren , J. Zeng , Z. Bin , F. Wang , R. Chen , J. Liu , X. Xia , P. Zhang , Sci. Rep. 2016, 6, 34956. 27734842 10.1038/srep34956 PMC5062118

[63] D. Boucher , T. Vu , A. L. Bain , M. Tagliaro‐Jahns , W. Shi , S. W. Lane , K. K. Khanna , FASEB J 2015, 29, 3326. 25917330 10.1096/fj.14-269944

[64] D. R. Wu , K. L. Gu , J. C. Yu , X. Fu , X. W. Wang , W. T. Guo , L. Q. Liao , H. Zhu , X. S. Zhang , J. Hui , Y. Wang , EMBO Rep. 2018, 19. 10.15252/embr.201745657 PMC5989781 29735517

[65] C. R. Sibley , Biochem. Soc. Trans. 2014, 42, 1196. 25110025 10.1042/BST20140102

[66] Z. Lin , P. J. Hsu , X. Xing , J. Fang , Z. Lu , Q. Zou , K. J. Zhang , X. Zhang , Y. Zhou , T. Zhang , Y. Zhang , W. Song , G. Jia , X. Yang , C. He , M. H. Tong , Cell Res. 2017, 27, 1216. 28914256 10.1038/cr.2017.117 PMC5630681

[67] J. Koubova , D. B. Menke , Q. Zhou , B. Capel , M. D. Griswold , D. C. Page , Proc. Natl. Acad. Sci. U S A 2006, 103, 2474. 16461896 10.1073/pnas.0510813103 PMC1413806

