# Supplementary material for: CWF19L2 is Essential for Male Fertility and Spermatogenesis by Regulating Alternative Splicing
Source: Adv Sci (Weinh). 2024 Jun 18;11(31):2403866. doi: 10.1002/advs.202403866 (PMC11336944; doi:10.1002/advs.202403866)
Supplement: Supplementary file 1 — Supporting Information [file ADVS-11-2403866-s001.docx]

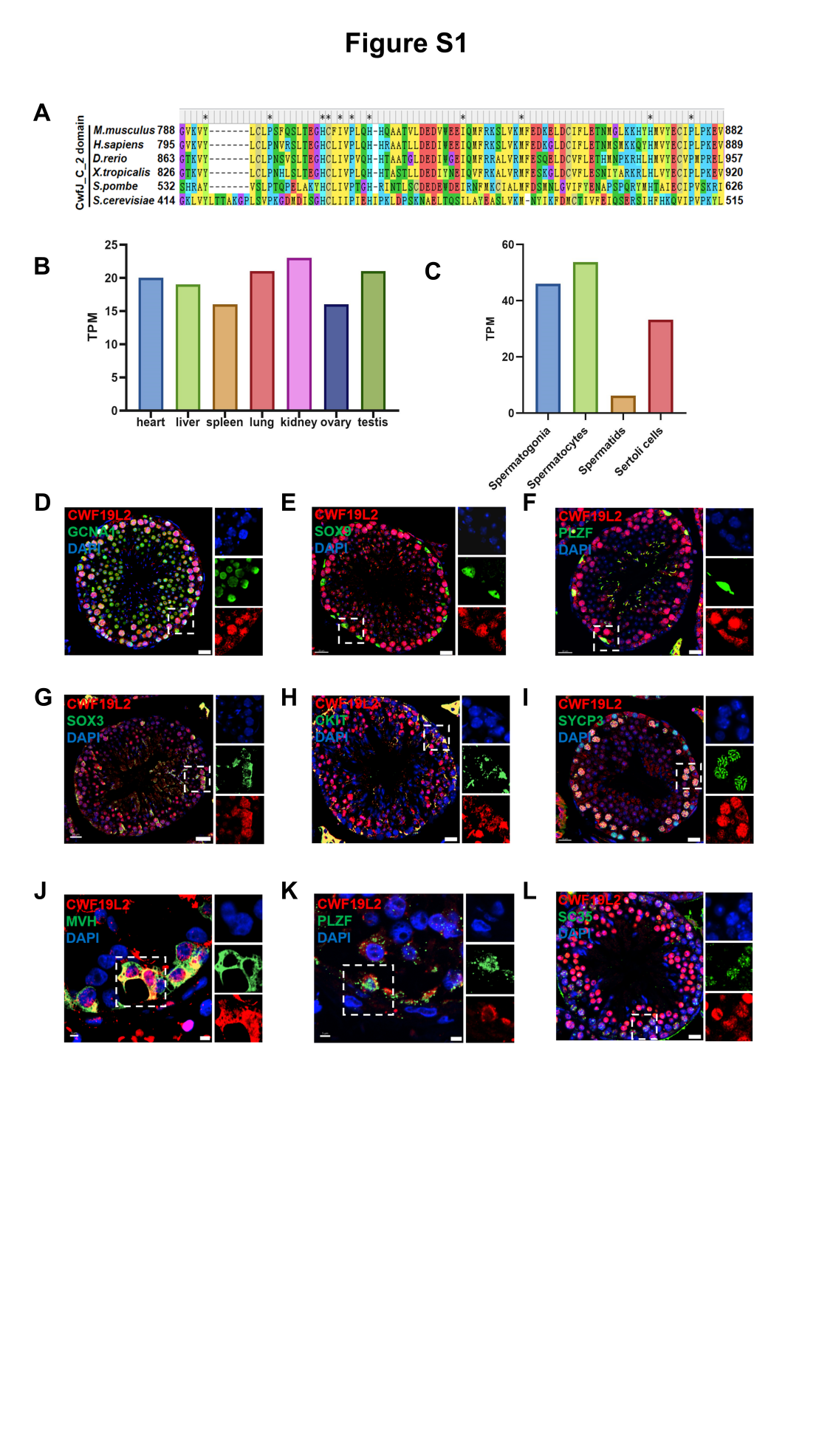


**Figure S1. Expression pattern of *Cwf19l2* during spermatogenesis**

1. Multiple sequence alignment of C-terminal CwfJ domain showed evolutionary conservation of CWF19L2. Essential evolutionarily conserved residues were marked with [asterisk](file:///C:\Users\王诗语\Desktop\CWF-投稿版-V1\4-SA\Supplementary%20Data%201-FigS1-S10.docx#asterisk)*.
2. C*wf19l2* mRNA expression levels of multiple organs in adult WT mice from Expression Atlas.
3. C*wf19l2* mRNA expression levels in spermatogonia, spermatocytes, spermatids, and Sertoli cells from the FANTOM5 project.
4. Co-immunofluorescence staining of the germ cell marker GCNA1 (green) with CWF19L2(red) in testis sections of adult WT mice. DNA was stained with DAPI. Scale bars = 15 µm
5. Co-immunofluorescence staining of the Sertoli cell marker SOX9 (green) with CWF19L2(red) in testis sections of adult WT mice. DNA was stained with DAPI. Scale bars = 20 µm.
6. Co-immunofluorescence staining of the undifferentiated spermatogonia marker PLZF (green) with CWF19L2(red) in testis sections of adult WT mice. DNA was stained with DAPI. Scale bars = 20 µm.
7. Co-immunofluorescence staining of the progenitor spermatogonia marker SOX3 (green) with CWF19L2(red) in testis sections of adult WT mice. DNA was stained with DAPI. Scale bars = 20 µm.
8. Co-immunofluorescence staining of the differentiated spermatogonia marker c-KIT (green) with CWF19L2(red) in testis sections of adult WT mice. DNA was stained with DAPI. Scale bars = 15 µm.
9. Co-immunofluorescence staining of the spermatocyte marker SYCP3 (green) with CWF19L2(red) in testis sections of adult WT mice. DNA was stained with DAPI. Scale bars = 15 µm.
10. Co-immunofluorescence staining of the germ cell marker MVH (green) with CWF19L2(red) in testis sections of adult human. DNA was stained with DAPI. Scale bars = 5 µm
11. Co-immunofluorescence staining of the Sertoli cell marker SOX9 (green) with CWF19L2(red) in testis sections of adult human. DNA was stained with DAPI. Scale bars = 5 µm.
12. Co-immunofluorescence staining of the nuclear speckles marker SC35 (green) with CWF19L2(red) in testis sections of adult WT mice. DNA was stained with DAPI. Scale bars = 15 µm

**
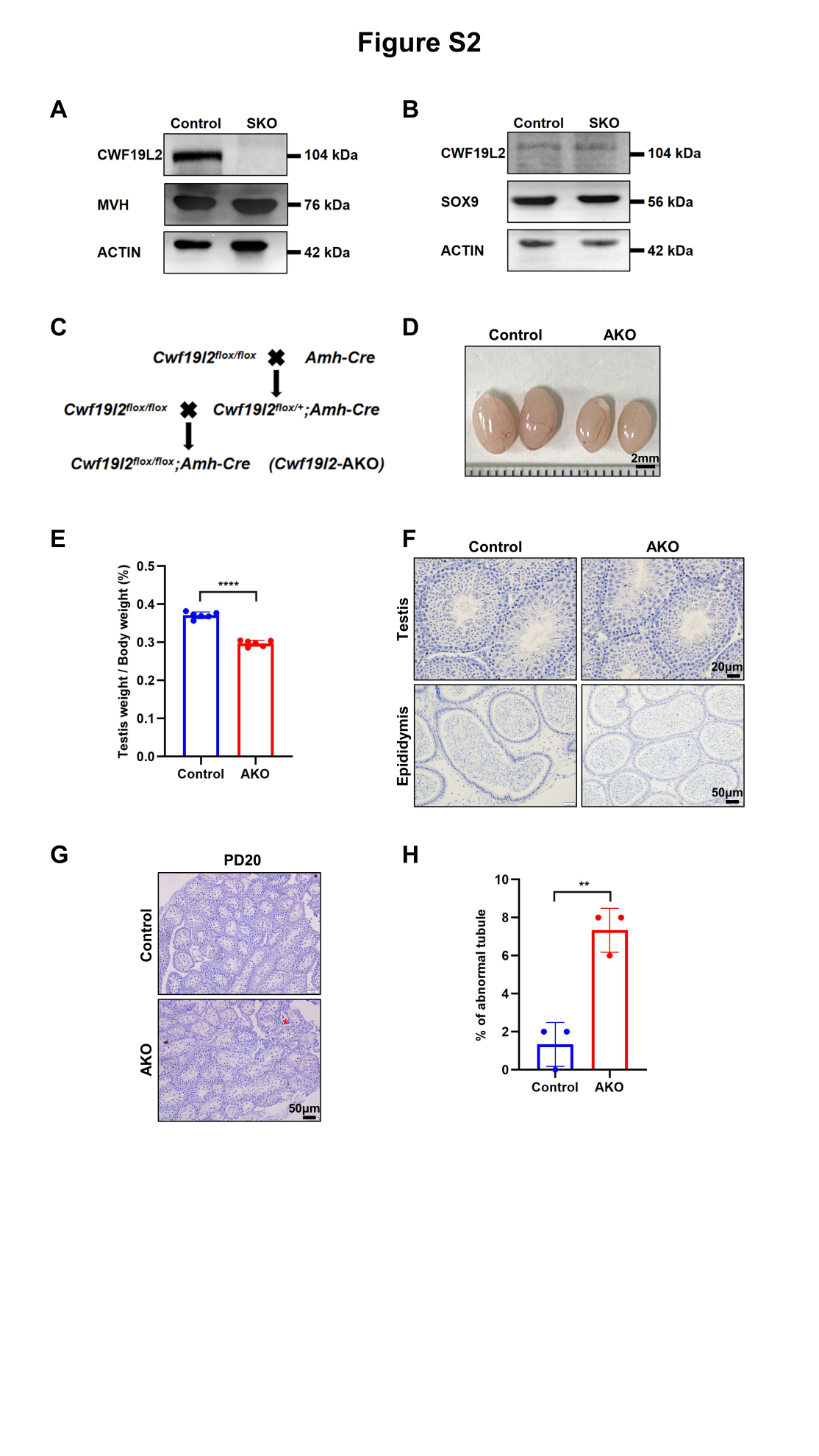
**

**Figure S2. Investigating the function of CWF19L2 in Sertoli cells using *Amh-Cre* mice**

1. Immunoblotting analysis of the knockout efficiency of CWF19L2 in fractions of spermatogenic cells in adult *Cwf19l2*-SKO and control mice. The germ cell marker MVH were used as indicators of enrichment efficiency. ACTIN served as the loading control.
2. Immunoblotting analysis of the knockout efficiency of CWF19L2 in fractions of somatic cells in adult *Cwf19l2*-SKO and control mice. The Sertoli cell marker SOX9 were used as indicators of enrichment efficiency. ACTIN served as the loading control.
3. Schematic for the generation of the *Cwf19l2* conditional knockout in Sertoli cells using *Amh-Cre.*
4. Gross morphology of the testes of adult *Cwf19l2*-AKO and control mice. Scale bars = 2 mm.
5. The ratio of testis weight to body weight in adult *Cwf19l2*-AKO and control mice. Data are presented as the mean ± SD, n = 6, *****P* < 0.0001 by two-tailed Student’ s *t*-test.
6. Hematoxylin staining of testes (top panel, scale bars = 20 µm) and epididymis (bottom panel, scale bars = 50 µm) of adult *Cwf19l2*-AKO and control mice.
7. Hematoxylin staining of testes of PD20 *Cwf19l2*-AKO and control mice. Asterisks, vacuolized and atrophied seminiferous tubules. Scale bars = 50 µm.
8. The percentage of abnormal (vacuolized and/or atrophied) seminiferous tubules in testes of PD20 *Cwf19l2*-AKO and control mice. Data are presented as the mean ± SD, n = 3, ***P* < 0.01 by two-tailed Student’ s *t*-test.

**
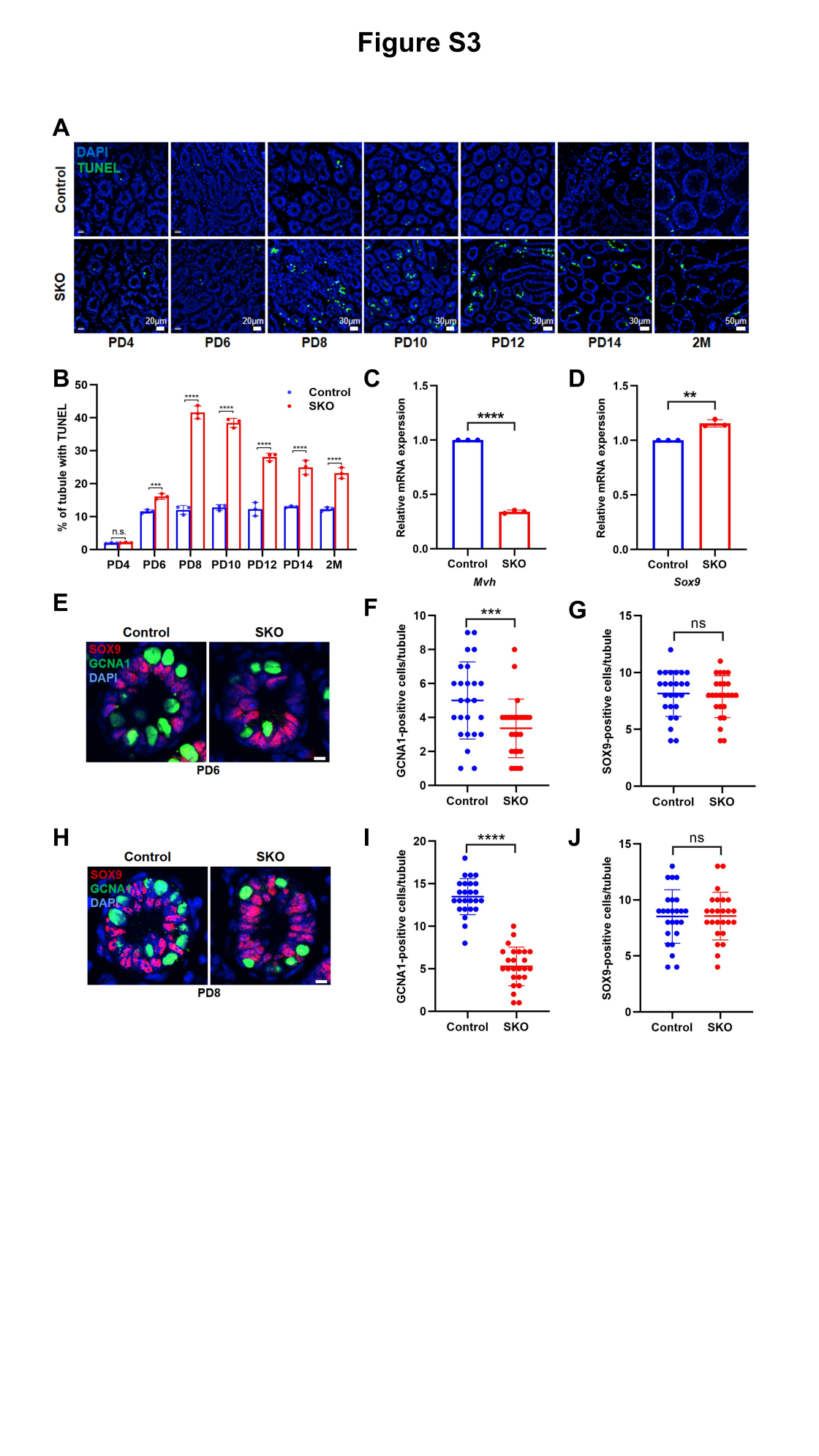
**

**Figure S3. The germ cells significantly decreased in *Cwf19l2*-SKO testes**

1. TUNEL analysis in different developmental stages of *Cwf19l2*-SKO and control testes. The DNA was stained with DAPI. Scale bars = 20 µm.
2. The quantification of TUNEL-positive tubule in (C). Data are presented as the mean ± SD, n = 3, ns: not significant, ****P* < 0.001, *****P* < 0.0001 by two-tailed Student’ s *t*-test.
3. QPCR analysis of *Mvh* mRNA levels in germ cells from *Cwf19l2*-SKO and control mice. Data are presented as mean ± SD, n = 3, *****P* < 0.0001 by two-tailed Student’ s *t*-test.
4. QPCR analysis of *Sox9* mRNA levels in germ cells from *Cwf19l2*-SKO and control mice. Data are presented as mean ± SD, n = 3, ***P* < 0.01by two-tailed Student’ s *t*-test.
5. Co-immunofluorescence staining of GCNA1(green) with SOX9(red) in testis sections of PD6 *Cwf19l2*-SKO and control mice. DNA was stained with DAPI. Scale bars = 5 µm.
6. The quantification of GCNA1-positive cells per tubule in (G). Data are presented as the mean ± SD, ****P* <0.001 by two-tailed Student’ s *t*-test.
7. The quantification of SOX9-positive cells per tubule in (G). Data are presented as the mean ± SD, ns: not significant by two-tailed Student’ s *t*-test.
8. Co-immunofluorescence staining of GCNA1(green) with SOX9(red) in testis sections of PD8 *Cwf19l2*-SKO and control mice. DNA was stained with DAPI. Scale bars = 5 µm.
9. The quantification of GCNA1-positive cells per tubule in (J). Data are presented as the mean ± SD, *****P* <0.0001 by two-tailed Student’ s *t*-test.
10. The quantification of SOX9-positive cells per tubule in (J). Data are presented as the mean ± SD, ns: not significant by two-tailed Student’ s *t*-test.


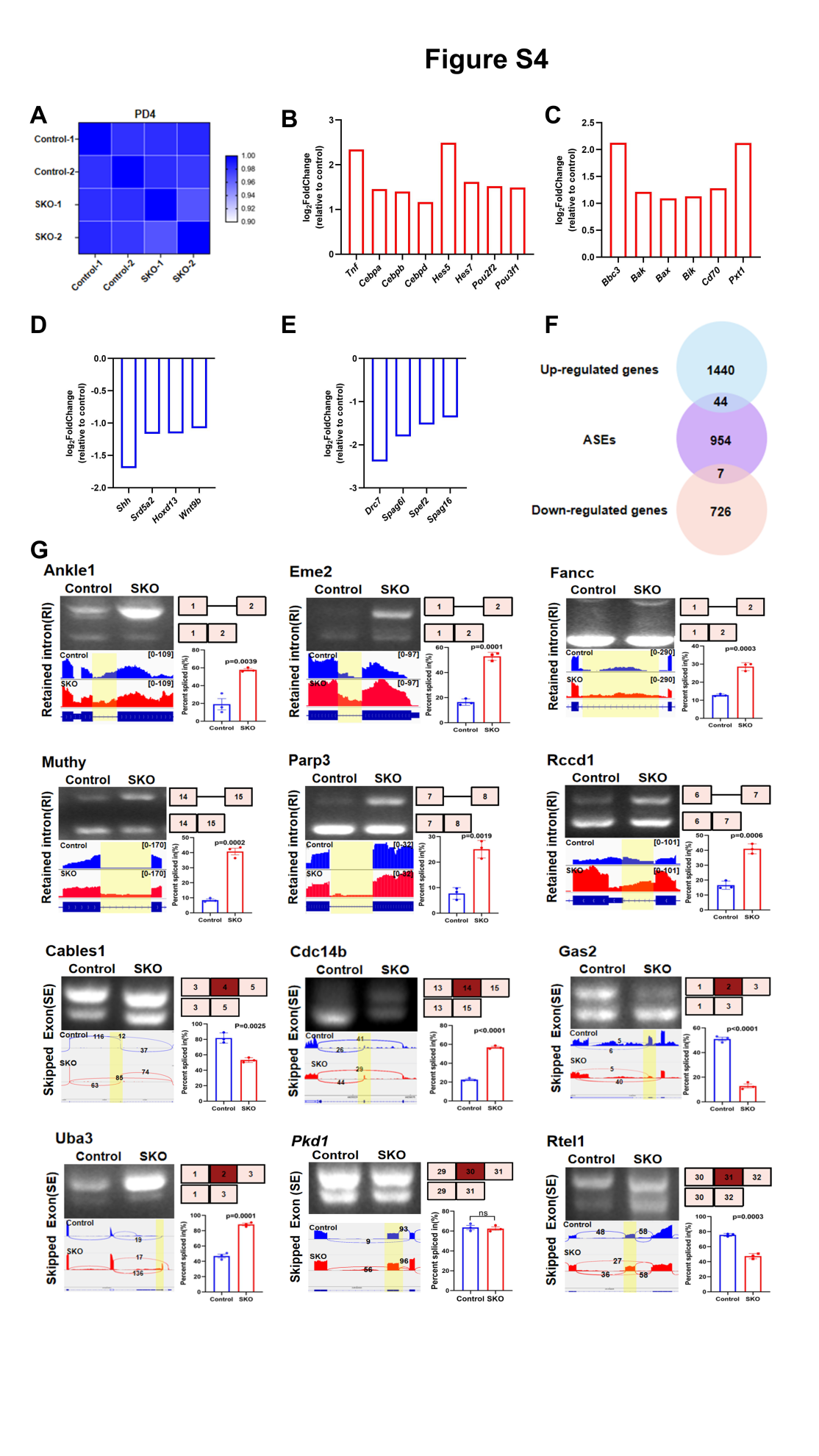


**Figure S4. Effects of CWF19L2 on splicing and transcription during spermatogenesis**

1. Pearson correlation in the transcriptomes of PD4 testes in *Cwf19l2*-SKO and control mice.
2. Upregulated DEGs determined by RNA-seq analysis related to regulation of transcription.
3. Upregulated DEGs determined by RNA-seq analysis related to apoptosis.
4. Downregulated DEGs determined by RNA-seq analysis related to male genitalia development.
5. Downregulated DEGs determined by RNA-seq analysis related to sperm axoneme assembly.
6. Venn diagram showing the common genes between DEGs and ASEs.
7. Visualization and validation of abnormal ASEs in germ cells from *Cwf19l2*-SKO and control mice.


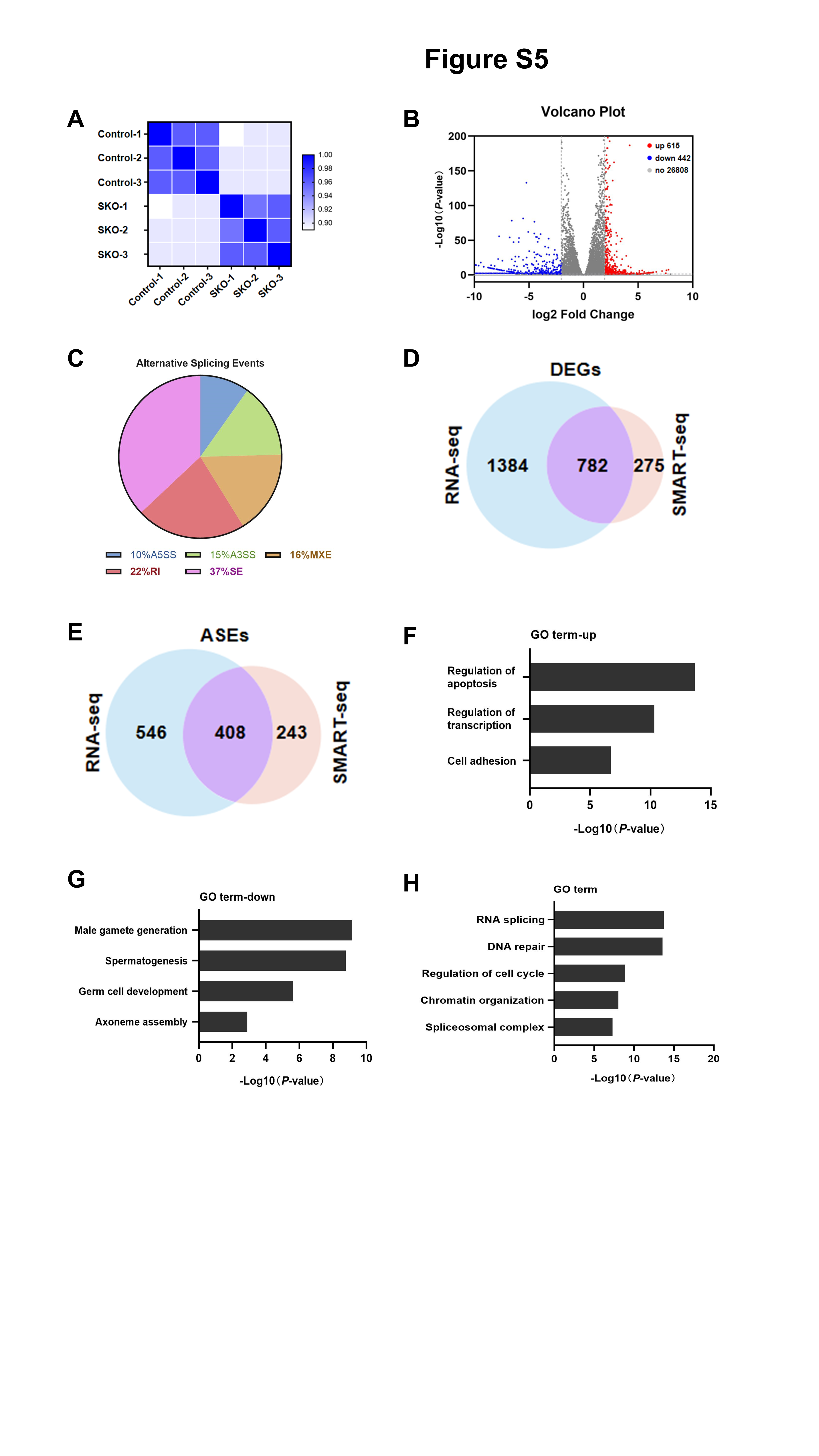


**Figure S5: SMART-seq in c-KIT positive spermatogeonia of *Cwf19l2*-SKO and control mice**

1. Pearson correlation in the transcriptomes of c-KIT positive spermatogeonia of *Cwf19l2*-SKO and control mice.
2. Volcano plot of DEGs determined by SMART-seq in c-KIT positive spermatogeonia of *Cwf19l2*-SKO and control mice. Blue dots represent significantly downregulated genes, red dots represent significantly upregulated genes, and gray dots represent unchanged genes.
3. Pie chart depicting the proportions of different types of ASEs in the SMART-seq data.
4. Venn diagram showing the shared DEGs between SMART-seq and RNA-seq.
5. Venn diagram showing the shared ASEs between SMART-seq and RNA-seq.
6. GO analysis of the significantly upregulated genes in the SMART-seq data.
7. GO analysis of the significantly downregulated genes in the SMART-seq data
8. GO analysis of genes with abnormal ASEs in the SMART-seq data.


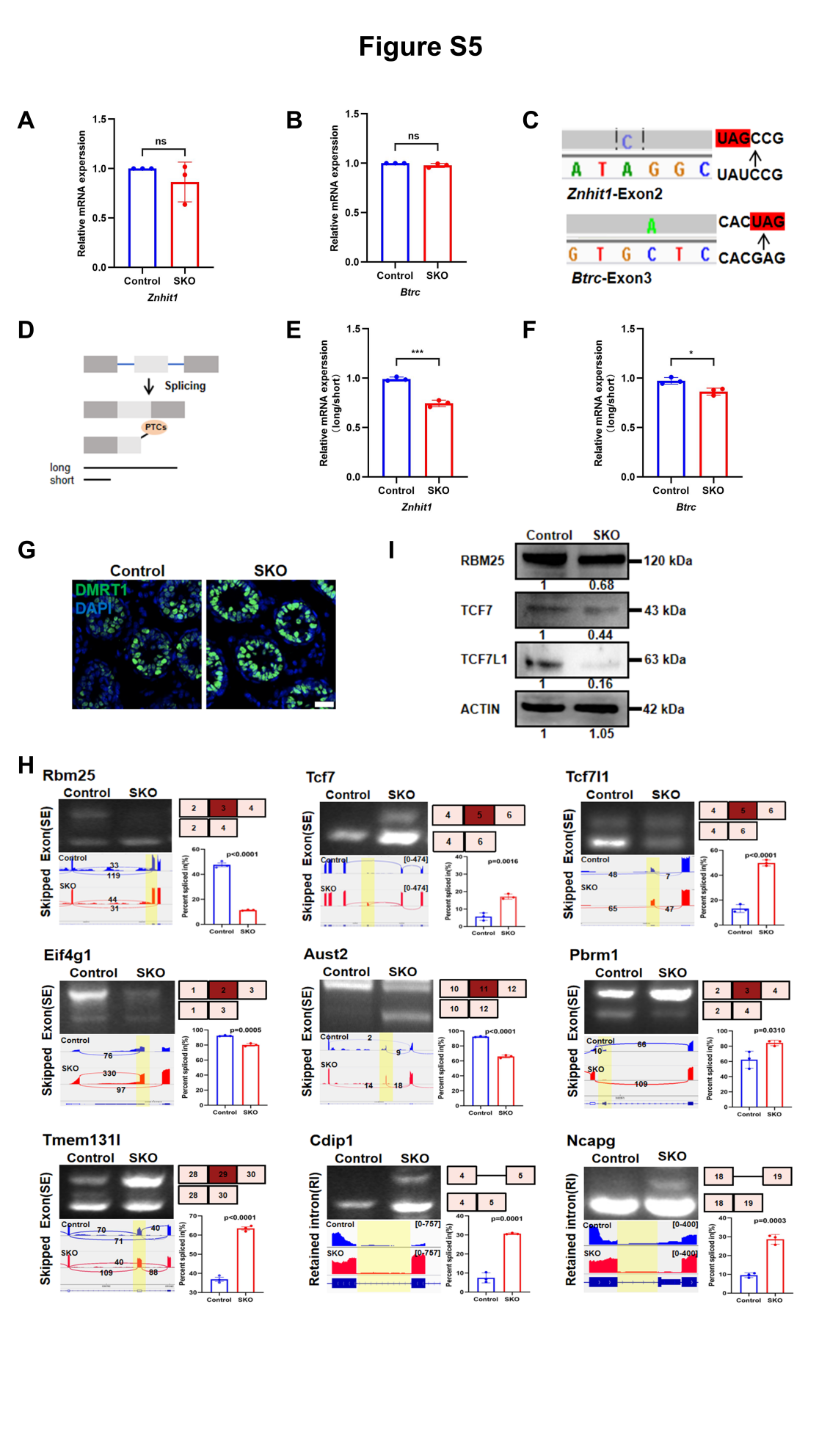


**Figure S6. Deletion of CWF19L2 in germ cells caused blocked spermatogenesis**

1. QPCR analysis of *Znhit1* mRNA levels in germ cells sorted from *Cwf19l2*-SKO and control mice. Data are presented as means ± SD; n = 3; ns, not significant by two-tailed Student’ s *t*-test.
2. QPCR analysis of *Btrc* mRNA levels in sorted germ cells from *Cwf19l2*-SKO and control mice. Data are presented as means ± SD; n = 3; ns, not significant by two-tailed Student’ s *t*-test.
3. Correct and mutant sequences in skipped exon 2 of *Znhit1* and exon 3 of *Btrc* (left) leading to premature termination codons (red highlight, right).
4. Schematic for design of short and long primers targeting sequence immediately upstream of the skipped exon to amplify transcripts containing the skipped exon (long) or not (short) by qPCR.
5. QPCR analysis of the ratio of long-to-short isoforms of *Znhit1* in sorted germ cells of *Cwf19l2*-SKO and control mice. Data are presented as means ± SD, n = 3, ****P* < 0.001 by two-tailed Student’ s *t*-test.
6. QPCR analysis of the long-to-short isoform ratio for *Btrc* in sorted germ cells of *Cwf19l2*-SKO and control mice. Data are presented as means ± SD, n = 3, **P* < 0.05 by two-tailed Student’ s *t*-test.
7. Immunofluorescence staining of DMRT1 (green) in testis sections of PD10 *Cwf19l2*-SKO and control mice. DNA was stained with DAPI. Scale bars = 15 µm.
8. Visualization and validation of abnormal ASEs related to spermatogenesis in germ cells from *Cwf19l2*-SKO and control mice.
9. Immunoblotting analysis of RBM25, TCF7, TCF7L1 protein in testes of PD10 *Cwf19l2*-SKO and control mice. ACTIN served as the loading control.


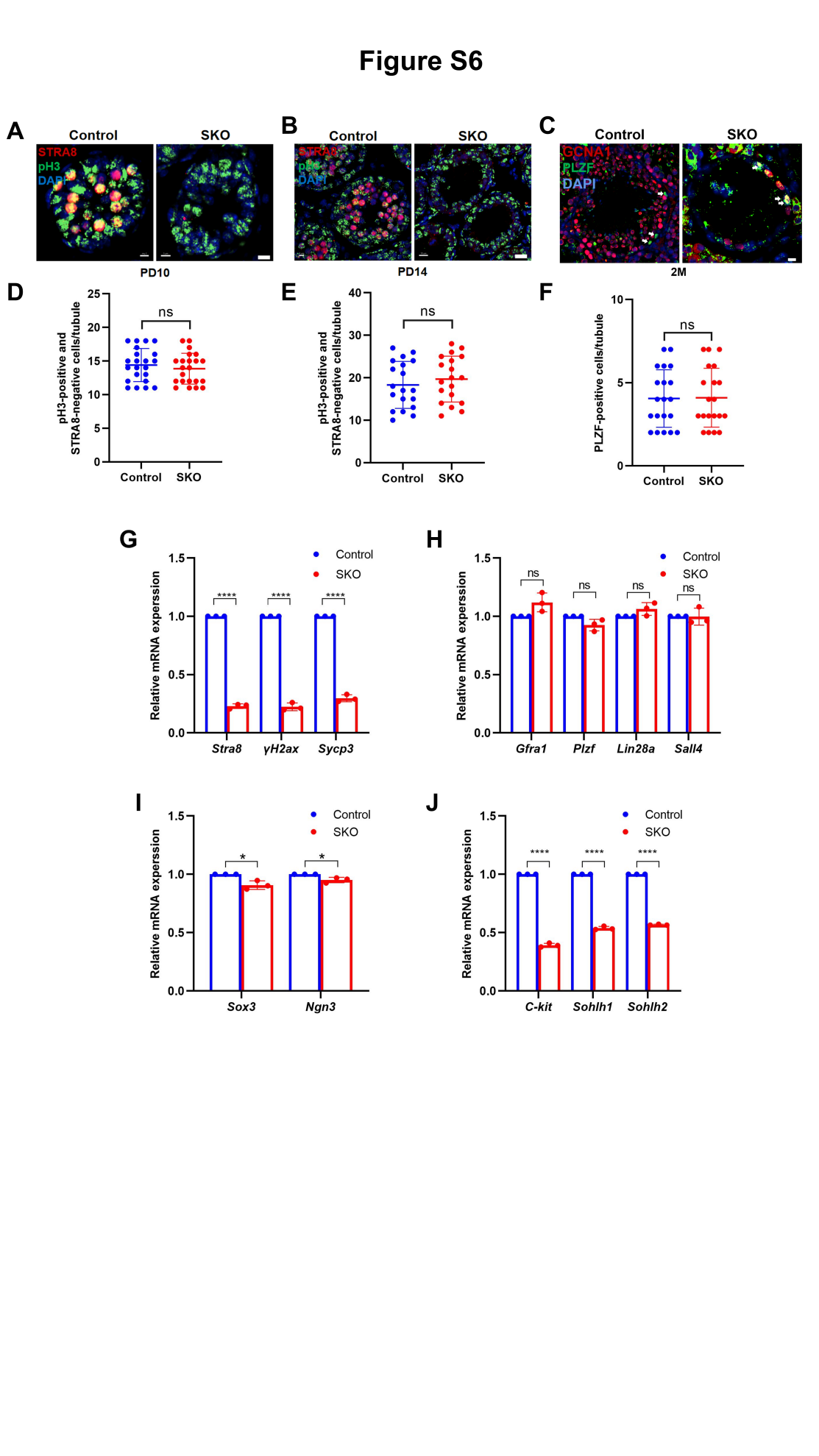


**Figure S7. Spermatogonia without CWF19L2 could self-renew but not differentiate**

1. Co-immunofluorescence staining of mitotic and meiotic metaphase cells marker pH3 (green) with meiotic initiation marker STRA8(red) in testis sections of PD10 *Cwf19l2*-SKO and control mice. DNA was stained with DAPI. Scale bars = 5 µm.
2. Co-immunofluorescence staining of the mitotic and meiotic metaphase cell marker pH3 (green) with the meiotic initiation marker STRA8 (red) in testis sections of PD14 *Cwf19l2*-SKO and control mice. DNA was stained with DAPI. Scale bars = 5 µm.
3. Co-immunofluorescence staining of GCNA1 (red) with PLZF (green) in testis sections of adult *Cwf19l2*-SKO and control mice. DNA was stained with DAPI. Scale bars = 15 µm.
4. The quantification of pH3-positive and STRA8-negative cells per tubule in (A). Data are presented as the mean ± SD, ns: not significant by two-tailed Student’ s *t*-test.
5. The quantification of pH3-positive and STRA8-negative cells per tubule in (B). Data are presented as the mean ± SD, ns: not significant by two-tailed Student’ s *t*-test.
6. The quantification of PLZF-positive cells per tubule in (C). Data are presented as the mean ± SD, ns: not significant by two-tailed Student’ s *t*-test.
7. QPCR analysis of the mRNA levels of the meiosis-related genes *Stra8*, *γH2ax*, and *Sycp3* in germ cells from *Cwf19l2*-SKO and control. Data are presented as mean ± SD, n = 3, *****P* < 0.0001 by two-tailed Student’ s *t*-test.
8. QPCR analysis of the mRNA levels of SSCs self-renewal-related and SSCs maintenance-related genes *Gfra1, Plzf, Lin28a,* and *Sall4* in germ cells from *Cwf19l2*-SKO and control mice. Data are presented as mean ± SD, n = 3, ns: not significant by two-tailed Student’ s *t*-test.
9. QPCR analysis of the mRNA levels of progenitor-related genes *Sox3* and *Ngn3* in germ cells from *Cwf19l2*-SKO and control mice. Data are presented as mean ± SD, n = 3, **P* < 0.05 by two-tailed Student’ s *t*-test.
10. QPCR analysis of the mRNA levels of the spermatogina differentiation-related genes *c-Kit, Sohlh1,* and *Sohlh2* in sorted germ cells from *Cwf19l2*-SKO and control mice. Data are presented as the mean ± SD, n = 3, *****P* < 0.0001 by two-tailed Student’ s *t*-test.


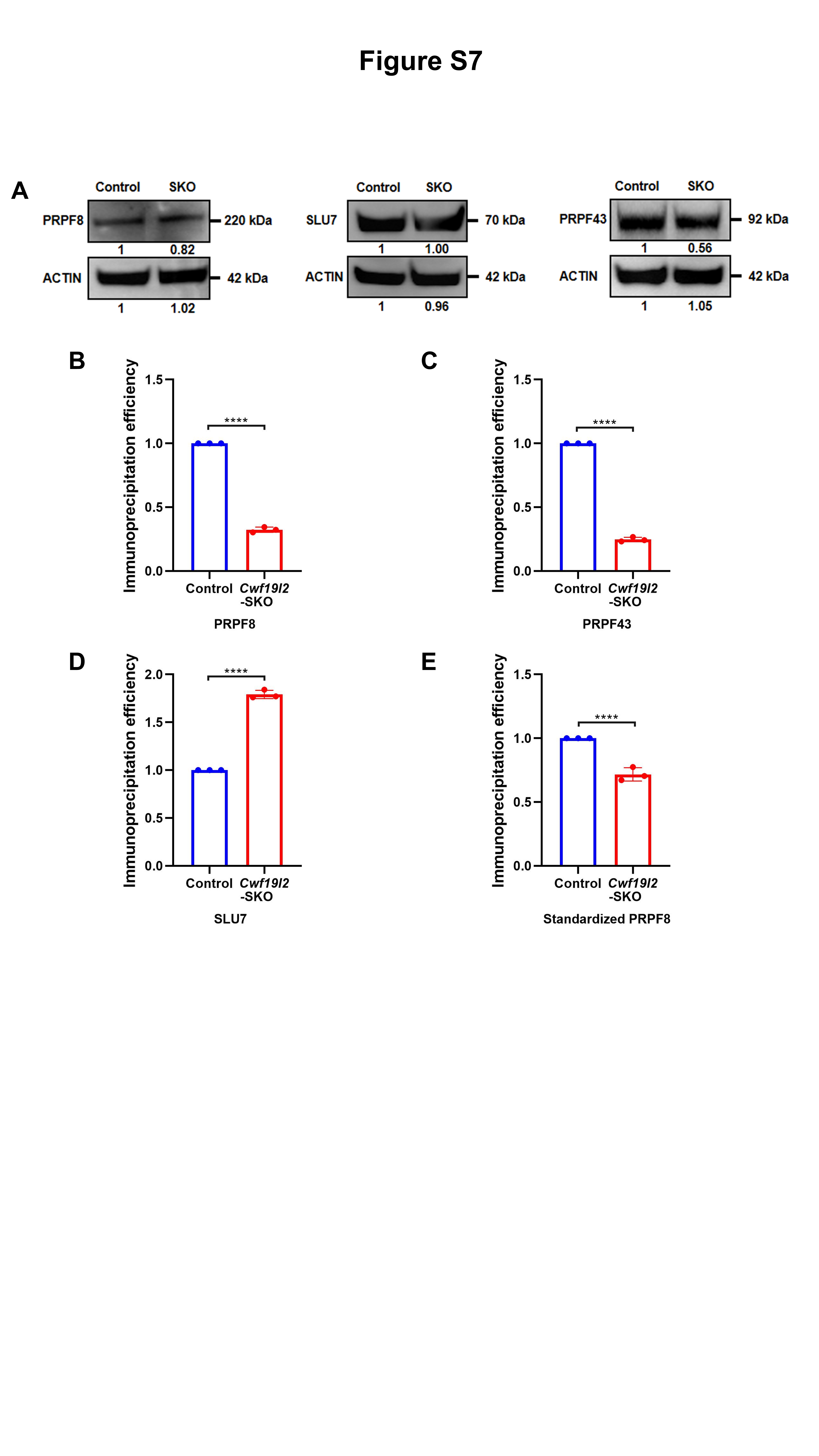


**Figure S8: Co-immunoprecipitation of PRPF8 interaction partners using PRPF8 antibody**

1. Immunoblotting analysis of PRPF8, SLU7, and PRPF43 protein in testes of PD6 *Cwf19l2*-SKO and control mice. ACTIN served as the loading control.
2. Relative immunoprecipitation efficiency of PRPF8 in sorted germ cells of *Cwf19l2*-SKO and control mice. Data are presented as mean ± SD, n = 3, *****P* < 0.0001 by two-tailed Student’ s *t*-test.
3. Relative immunoprecipitation efficiency of PRPF43 in sorted germ cells of *Cwf19l2*-SKO and control mice. Data are presented as mean ± SD, n = 3, *****P* < 0.0001 by two-tailed Student’ s *t*-test.
4. Relative immunoprecipitation efficiency of SLU7 in sorted germ cells of *Cwf19l2*-SKO and control mice. Data are presented as mean ± SD, n = 3, *****P* < 0.0001 by two-tailed Student’ s *t*-test.
5. Relative immunoprecipitation efficiency of PRPF43 in sorted germ cells with assuming equivalent levels of PRPF8 between WT and *Cwf19l2*-SKO samples. Data are presented as mean ± SD, n = 3, *****P* < 0.0001 by two-tailed Student’ s *t*-test.


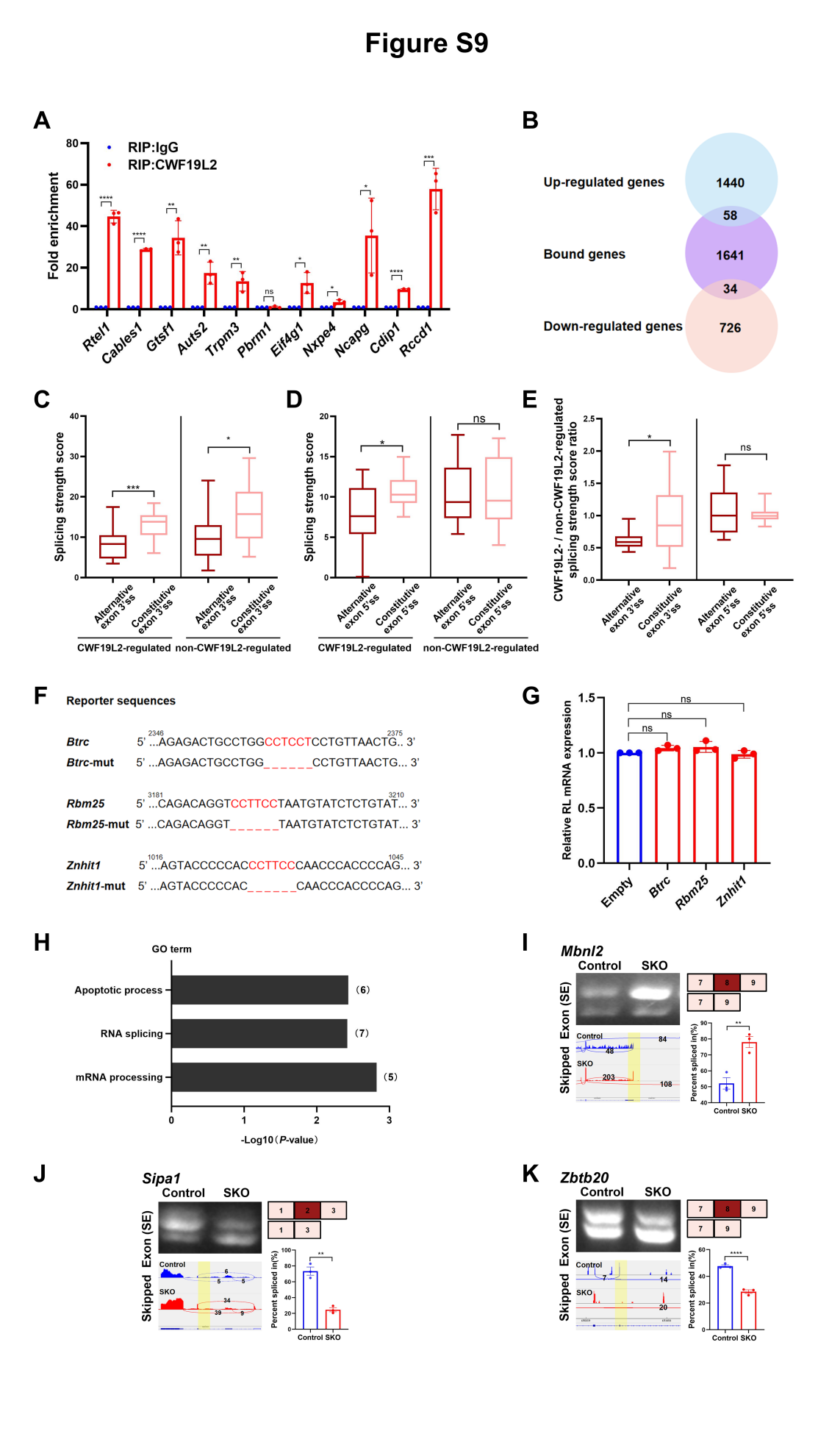


**Figure S9. CWF19L2 regulates alternative splicing directly and indirectly**

1. Histograms showing RIP-qPCR analysis of some selected mRNA of 141 common genes by anti-CWF19L2 antibody and control IgG. Data were presented as mean ± SD, n = 3, ns: not significant, **P* < 0.05, ***P* < 0.01, ****P* < 0.001, *****P* < 0.0001 by two-tailed Student’ s *t*-test.
2. Venn diagram showing the common genes between CWF19L2 bound genes and DEGs.
3. Box plots of splicing strength scores for 3’ splice sites in CWF19L2-regulated and non-CWF19L2-regulated skipped exons. ****P* < 0.001, **P* < 0.05 by two-tailed Student’ s *t*-test.
4. Box plots of splicing strength scores for 5’ splice sites in CWF19L2-regulated and non-CWF19L2-regulated skipped exons. **P* < 0.05, ns: not significant by two-tailed Student’ s *t*-test.
5. Box plots of splicing strength score ratio of CWF19L2-regulated to non-CWF19L2-regulated skipped exons. **P* < 0.05, ns: not significant by two-tailed Student’ s *t*-test.
6. Predicted CWF19L2-binding motifs and sequences of *Btrc*, *Rbm25* and *Znhit1* and mutated luciferase reporter.
7. QPCR analysis of the mRNA levels of luciferase reporters. Data are presented as mean ± SD, n = 3, ns: not significant by two-tailed Student’ s *t*-test.
8. GO term enrichment analysis of common genes with abnormal ASEs between RNA-seq of CWF19L2 and RBFOX1.
9. Visualization and validation of *Mbnl2* abnormal ASEs in germ cells from *Cwf19l2*-SKO and control mice.
10. Visualization and validation of *Sipa1* abnormal ASEs in germ cells from *Cwf19l2*-SKO and control mice.
11. Visualization and validation of *Zbtb20* abnormal ASEs in germ cells from *Cwf19l2*-SKO and control mice.


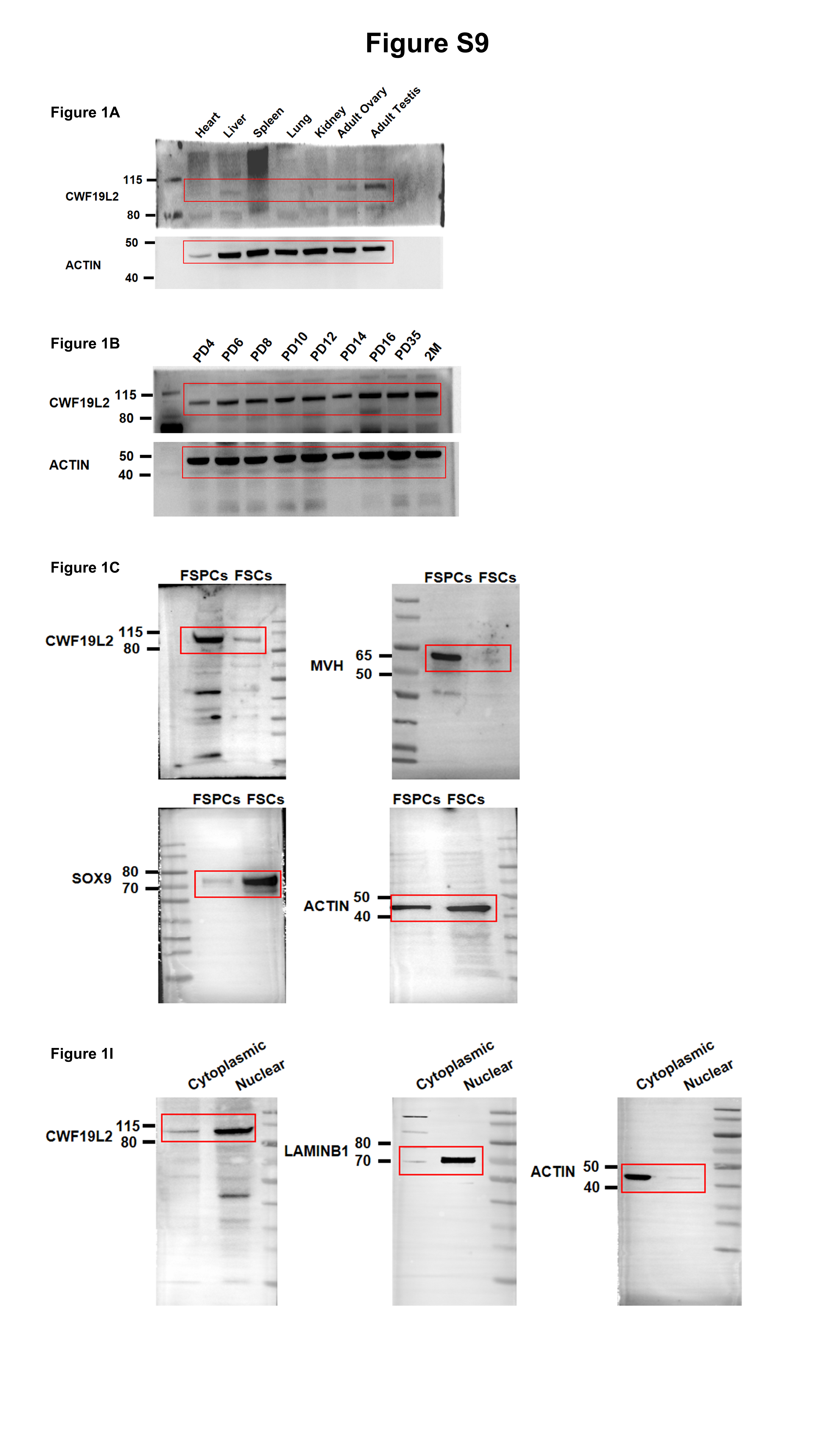


**Figure S10. Uncropped western blot gel images in Figure 1A, 1B, 1C, 1I**

Red boxes highlight lanes used in figures.

**
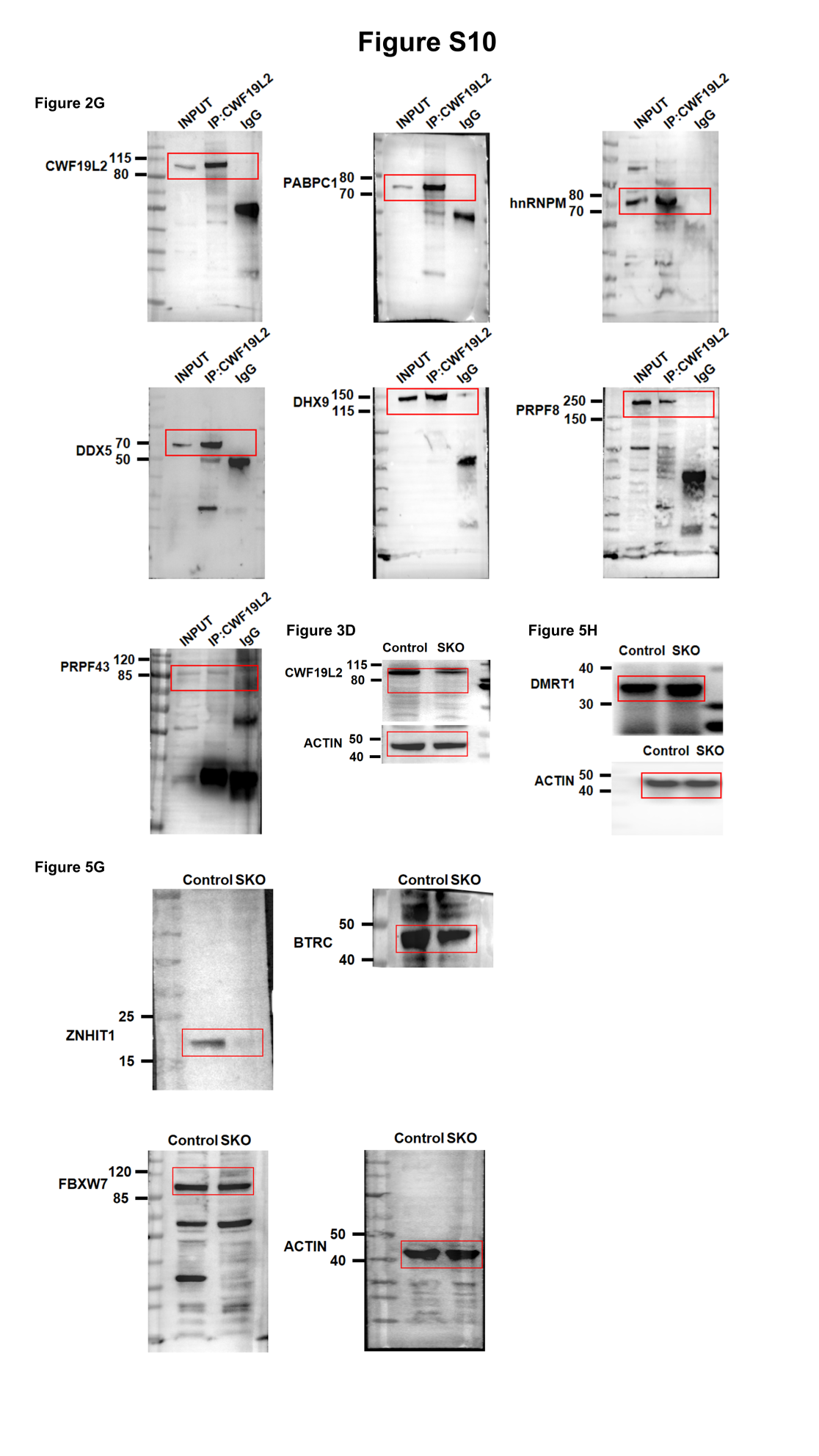
**

**Figure S11. Uncropped western blot gel images in Figure 2G, 3D, 5G, 5H**

Red boxes highlight lanes used in figures.

**
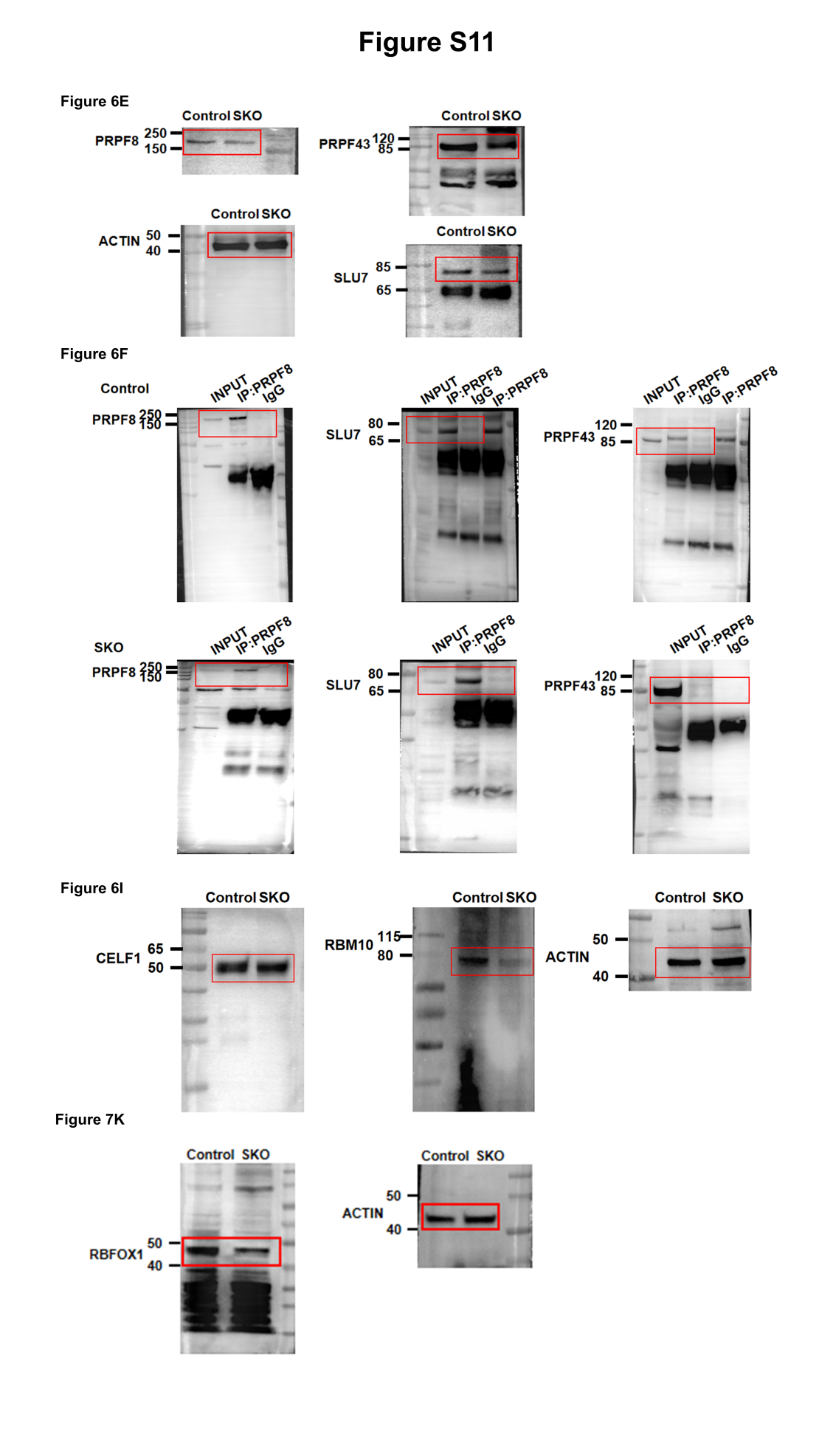
**

**Figure S12. Uncropped western blot gel images in Figure 6E, 6F, 6I, 7K**

Red boxes highlight lanes used in figures.
